# Relationship Between Inflammatory Readings and the Degree of Coronary Atherosclerosis (Pilot Study)

**DOI:** 10.3390/jcm14010122

**Published:** 2024-12-28

**Authors:** Agnė Liuizė (Abramavičiūtė), Aušra Mongirdienė, Jolanta Laukaitienė

**Affiliations:** Department of Biochemistry, Lithuanian University of Health Sciences, LT 44307 Kaunas, Lithuania; ausra.mongirdiene@lsmu.lt (A.M.);

**Keywords:** total blood count readings, systemic immune-inflammation index, coronary artery disease, atherosclerosis, SIRI

## Abstract

**Background/Objectives:** Some calculated total blood count readings are investigated as novel additional readings to help with evaluation of personalized CAD patients’ clinical management and prognosis. We aimed to investigate the association between readings such as NLR, MLR, PLR, NMR, LMR, MHR, SII, and SIRI and the severity of CAD in patients with SAP. **Methods:** This retrospective pilot study included 166 patients. All patients underwent CA or CCTA, or both, to assess severity of CAD. Patients were divided three ways: (1) according to presence (*n* = 146) or absence (*n* = 20) of CAD; (2) according to Gensini score; (3) according to the CAD-RADS score. **Results**: Patients with CAD had lower LMR, higher NLR, SIRI, MLR, and SII compared to patients without CAD *(p* < 0.001 and *p* = 0.018, respectively for SII). According to the CAD severity by Gensini score, the NLR, MLR, SII, and SIRI values increase and LMR decreases gradually with severity of CAD (*p* < 0.001). A moderate correlation was found between SII (*r* = 0.511, *p* < 0.001), NLR (*r* = 0.567, *p* < 0.001), and SIRI (*r* = 0.474, *p* < 0.001) and severity of CAD according to Gensini score. MLR and LMR had a low corelation with severity of CAD according to Gensini score (*r* = 0.356, *p* < 0.001; *r* = −0.355, *p* < 0.001, respectively). The CAD-RADS score weakly correlated with NLR and MHR *(r* = 0.365, *p* < 0.001; *r* = 0.346, *p* < 0.001, respectively), and moderately with LMR, MLR, and SIRI (*r* = −0.454, *p* < 0.001; *r* = 0.455, *p* < 0.001; *r* = 0.522, *p* < 0.001, respectively). **Conclusions:** NLR, LMR, and SIRI appear to be potential predictors of chronic inflammation, and SIRI is the best predictor of the degree of atherosclerosis of all the other assessed blood parameters.

## 1. Introduction

Atherosclerosis is a chronic arterial disease involving vasoconstriction and activation of the inflammatory process leading to the formation of atherosclerotic plaque [1]. Atherosclerosis, as an inflammatory disease, is crucial for the onset and development of coronary artery disease (CAD) [2]. CAD remains the leading cause of mortality and morbidity worldwide. Therefore, the identification of high-risk patients with CAD is useful for clinical management and prognosis [3]. Some calculated total blood count readings are investigated as novel additional readings to help with evaluation of CAD patients’ condition, clinical management, and prognosis.

Markers of inflammatory processes, such as neutrophil-to-lymphocyte ratio (NLR) [4], monocyte-to-lymphocyte ratio (MLR) [5], and platelet-to-lymphocyte ratio (PLR) [6], have been shown to be associated with the severity of CAD and poor cardiovascular prognosis.

Studies have shown that immune and inflammatory reactions are closely linked to the development of atherosclerosis [7]. Recently, much attention has been paid to blood cell analysis as a routine laboratory test. Some of the most important cells of the immune system are lymphocytes, neutrophils, monocytes, and macrophages, which play different and important roles in the development of atherosclerosis. For instance, neutrophils can accelerate atherosclerosis at various stages, e.g., by activating macrophages, recruiting monocytes, and exerting cytotoxic effects, while lymphocytes modulate the inflammatory response, and thus have an anti-atherosclerotic effect [8,9]. Platelets adhering to the blood vessel wall have been shown to promote leukocyte aggregation and initiate the progression of atherosclerosis before leukocytes penetrate the atherosclerotic plaque [9,10].

It has been shown that neutrophil, lymphocyte, monocyte, and platelet counts may have a prognostic role in the development of CAD, and therefore, as mentioned above, the novel parameters NLR, MLR, and PLR were determined [4,5,6]. Many studies have been carried out to assess their evidence as a marker of subclinical inflammation. NLR and PLR were thought to be independent predictors of atherosclerosis severity, and high MLR values can help identify vulnerable plaques in patients with stable angina [3].

A growing number of experimental and clinical investigators confirm that the lymphocyte-to-monocyte ratio (LMR) plays a crucial role in chronic inflammation. Monocytes are pro-inflammatory and differentiate into macrophages in the event of endothelial dysfunction, which subsequently phagocytose lipids in the sub-endothelial space and may differentiate into mast cells and induce atherosclerotic plaque development [11]. Lymphocytes are thought to exert anti-inflammatory effects and regulate the inflammatory response in the pathogenesis of atherosclerosis by enhancing the immune response and are affected by serum catecholamine and cortisol levels during the systemic stress response [12]. Thus, LMR is involved in all stages of coronary atherosclerosis, from initial endothelial dysfunction and plaque disruption to acute atherothrombosis [13].

The neutrophil-to-monocyte ratio (NMR) is also found to be higher in patients with CAD compared to healthy individuals. This ratio is a novel marker of systemic inflammation and both neutrophils and monocytes play an important role in the development and progression of atherosclerosis, the main cause of CAD. Elevated NMR is even associated with CAD severity and can therefore be used as a prognostic indicator in these patients. In addition, NMR is generally lower in healthy people, suggesting a more balanced inflammatory response [14].

Monocyte-to-high-density lipoprotein cholesterol (HDL-C) ratio (MHR) is also presented as a low-cost composite prognostic indicator reflecting the balance between inflammatory and lipid metabolism. It is known that monocytes have the capacity to migrate into the subendothelial space and take up lipoproteins, and hypercholesterolaemia promotes their faster migration. In contrast, HDL-C molecules have anti-atherosclerotic properties due to their function in the so-called “reverse cholesterol transport”, which prevents monocyte activation and recruitment. Thus, MHR is also considered as a diagnostic indicator of the presence and severity of CAD [15].

Another novel and cheap reading is the systemic inflammatory response index (SIRI), which is calculated using even three types of white blood cells: neutrophils, lymphocytes, and monocytes. Studies have shown that the SIRI has a prognostic value in suspected CAD because it includes neutrophils, which have a tendency to activate macrophages and stimulate further recruitment of monocytes and cytotoxicity, accelerating all stages of atherosclerosis; lymphocytes, which regulate inflammation, and therefore have an anti-atherosclerotic effect, and monocytes, which promote the inflammatory process that leads to plaque formation, progression, and eventual rupture [16]. Li Y. and co-authors conducted a study to assess the association of SIRI with adverse cardiovascular prognosis in patients initially diagnosed with CAD. Their exclusion criteria were quite similar to ours (active tumor or paraneoplastic syndrome, acute infection, severe renal failure, severe hepatic failure, known inflammatory/autoimmune disease, active cerebrovascular disease), but the study included patients with diabetes mellitus [17]. Similarly, Urbanowicz T et al. attempted to assess the prognostic role of SIRI in the development of CAD in their study. They used few exclusion criteria (acute coronary syndromes, hematological and rheumatic diseases, or history of oncology) and included patients with other comorbidities, such as diabetes mellitus, chronic obstructive pulmonary disease, peripheral arterial disease, renal impairment, and history of stroke [16]. These conditions could have an influence on evaluated readings.

The systemic immune-inflammation index (SII) is presented as another important prognostic marker of CAD. It is calculated by multiplying the platelet count by the neutrophil-to-lymphocyte ratio [18]. The reading reflects the balance between inflammation and immune response. Candemir M. evaluated the association between SII and the severity of coronary atherosclerosis in patients diagnosed with SAP, with the same exclusion criteria as ours, but they also included patients with diabetes mellitus [3]. SII has recently been offered to be used for determination of the severity and prognosis of CAD, as it can provide a more detailed assessment of the inflammatory and immune status of patients with CAD [17,19].

Our study differs from previous studies in that it included patients with stable angina pectoris (SAP), as we tried to exclude patients with comorbidities that may have affected the laboratory measurements. Our study results should be useful in the future for a more accurate identifying of the SAP patients who are at the highest risk of significant coronary artery stenosis and who would benefit from more accurate differential diagnosis.

Therefore, this study aimed to investigate the relationship between estimated novel inflammatory markers and the severity of coronary atherosclerosis in patients with SAP with the possibility to use them with specific clinical relevance in predicting CAD severity.

## 2. Materials and Methods

### 2.1. Study Population

The retrospective pilot study included 166 eligible patients diagnosed with SAP and examined at the Cardiology Department of Kaunas Clinics between December 2022 and December 2023. The diagnosis of SAP was made according to the criteria set out in the guidelines [20]. Exclusion criteria were age less than 18 years, history of acute coronary syndrome, previous revascularization (previous coronary artery bypass grafting or percutaneous coronary intervention), peripheral arterial disease, chronic or acute heart failure, reduced left ventricular ejection fraction < 50%, severe valvular heart disease, acute or chronic infections, systemic inflammatory or autoimmune diseases, treatment with glucocorticoids in the last 3 months, and recent trauma or major surgery in the last month, as well as oncological, hematological, rheumatic, or endocrine diseases and hepatic or kidney failure (glomerular filtration rate < 60 mL/min.).

All patients underwent invasive coronary angiography (CA) or multi-slice computed tomographic coronary angiography (CCTA), or both, to assess coronary artery disease and its severity. This study is retrospective, so baseline characteristics and results of laboratory and other tests (echocardiography, CA, CCTA) were obtained from the hospital’s electronic medical records: age, sex, body mass index (BMI), heart rate, and systolic and diastolic blood pressure (BP), as well as comorbidities such as arterial hypertension (AH) and dyslipidemia, and risk factors such as smoking, obesity, early family history of cardiovascular disease, and use of medication (beta-blocker, statin, ezetimibe, angiotensin-converting enzyme (ACE) inhibitor or angiotensin II receptor blocker (ARB), calcium channel blocker (CaCB), mineralocorticoid receptor antagonist (MRA), antiplatelet agent (aspirin), trimetazidine, or ranolazine).

Coronary artery disease (CAD) refers to atherosclerotic lesions in the coronary arteries, and SAP is one of the symptoms experienced by people with CAD, which is characterized by a predictable feeling of chest discomfort or pain on exertion, which is alleviated with rest or with the use of medications [20]. In addition, symptoms of atypical angina, characterized by shortness of breath, abdominal distension, gas, abdominal pain, burning or tenderness in the back, shoulders, arms, or jaw, more common in women, were assessed and differentiated from other conditions which may mimic symptoms similar to those of stable angina, such as gastroenterological pathology (for example, gastro-esophageal reflux disease), musculoskeletal conditions (for example, straining of the muscles of the chest wall), lung disease, and anxiety or panic attacks. Patients with a history of acute coronary syndrome and previous revascularization, as mentioned before, were excluded.

All the investigations were approved and conducted in accordance with the guidelines of the local Bioethics Committee and adhered to the principles of the Declaration of Helsinki and Title 45, U.S. Code of Federal Regulations, Part 46, Protection of Human Subjects (revised 15 January 2009, effective 14 July 2009). This study was approved by the Regional Bioethics Committee at the Lithuanian University of Health Sciences (No.: BE-2-132, 16 December 2021).

### 2.2. Laboratory Measurements

Laboratory blood samples were taken before CA or CCTA. The results were obtained from patient histories: complete blood counts, total cholesterol, high-density lipoprotein cholesterol (HDL-C), low-density lipoprotein cholesterol (LDL-C), triglycerides, apolipoprotein B (apoB), and lipoprotein (a) (Lp(a)), as well as high-sensitivity C-reactive protein (hs-CRP) and uric acid. The following parameters were analyzed from the total blood count: leukocytes (neutrophils, lymphocytes, monocytes, eosinophils), erythrocytes, platelets, and mean platelet volume (MPV), and the ratio of these parameters were calculated: PLR was calculated by dividing platelet count by lymphocyte count, NLR by dividing neutrophil count to lymphocyte count, NMR as the ratio of neutrophil count to monocyte count, LMR as the ratio of lymphocyte count to monocyte count, MLR by dividing monocyte count to lymphocyte count, MHR as the ratio of monocytes to high-density lipoproteins, SIRI as MLR multiplied by neutrophils, and SII as NLR multiplied by platelets.

Arterial hypertension was defined as systolic and/or diastolic blood pressure ≥ 140 and/or 90 mmHg, respectively. The dyslipidemia group consisted of patients with total cholesterol concentration greater than 5 mmol/L or LDL-C concentration greater than 3 mmol/l or triglycerides concentrations greater than 1.7 mmol/L or HDL-C less than 1 mmol/l for men, <1.2 mmol/L for women, as well as patients who were already taking a statin, even though their LDL-C and triglycerides concentrations had already been lowered to within the normal limits. Smoking status was defined as current tobacco use. An early family history of cardiovascular disease was defined as early cardiovascular disease in first-degree relatives (age < 55 years in men and <65 years in women).

### 2.3. Instrumental Examination

We analyzed the following transthoracic echocardiography data from patient history: left ventricular measurements, such as left ventricular end-diastolic diameter and index (LVEDD, LVEDDi), thickness of intraventricular septal (IVS) and left ventricular posterior wall (LVPW), left ventricular mass (LVM) and left ventricular mass index (LVMi), and relative left ventricular wall thickness (RWT), as well as left ventricular ejection fraction (LVEF), left atrial diameter (LA), right atrial and right ventricular diameter (RA, RV), tricuspid annular systolic velocity (RV S′), and pulmonary artery systolic pressure (PASP). Also, left ventricular filling pressure measurements were taken, such as peak early diastolic velocity (E), late diastolic velocity (A), and E/A ratio to assess the degree of left ventricular diastolic dysfunction. Mitral annular early diastolic velocity (e′) and late diastolic velocity were measured in the septal (e′_sep_) and lateral (e′_lat_) mitral annulus, and the E/e′ ratio was calculated.

For patients with a low or intermediate probability of coronary artery disease, CCTA was selected based on the ESC 2019 guidelines for chronic coronary syndromes [20]. CCTA scans were performed on CT scanner (Canon Aquilion One Genesis Edition, Tustin, CA, USA) with a minimum of 64 slices. Pre-treatment with nitroglycerin was added according to the recommendations. If the patients’ pre-CCTA heart rate was higher than 60 bpm, heart rate control drugs such as beta-blockers were administered (sinus node I(f) channel inhibitor (ivabradine) was given to patients who could not take beta-blockers due to any contraindication).

Analysis of the scans was performed using a Vitrea workstation. Initially, the images were reconstructed at 75% of the cardiac cycle, with a thickness of 0.5 mm and 0.3 mm, respectively. The spatial resolution was 0.33 mm. In the presence of motion artefacts, additional reconstructions were performed at different time points of the R-R interval. CCTA scans were analyzed on site by a qualified radiologist and patients’ medical histories were concealed.

To determine the severity of coronary stenosis, CAD severity was categorized according to the Coronary Artery Disease Reporting and Data System (CAD-RADS) classification [21]. The CAD-RADS classification consisted of the following variants: CAD-RADS 0—no plaque or stenosis, CAD-RADS 1—minimal stenosis: 1 to 24%, CAD-RADS 2—mild stenosis: 25 to 49%, CAD-RADS 3—moderate stenosis: 50 to 69%, CAD-RADS 4—severe stenosis: 70 to 99%, or a left main ≥ 50%, or three-vessel obstructive disease ≥ 70%, CAD-RADS 5—at least one completely occluded coronary artery.

As recommended in the aforementioned ESC 2019 guidelines on chronic coronary syndromes [20], CA was performed in patients with a high clinical probability and severe symptoms that could not be treated with medication, or if obstructive CAD was suspected on CCTA scan.

CA was performed using standard Judkins methods through femoral artery or radial artery. Coronary angiograms were analyzed by experienced interventional cardiologist who were not informed about the patient’s clinical data. Coronary artery stenosis was judged by visual assessment. To assess the degree of atherosclerotic stenosis, the percentage degree of luminal narrowing was calculated by using the following formula: diameter of the normal vessel in the proximal part of stenotic vessel—diameter of stenotic site)/diameter of the proximal part of stenotic vessel × 100%.

The Gensini scoring system was used to assess the severity of CAD. The Gensini score was calculated for each patient from the coronary arteriogram, assigning a severity score for each form of coronary stenosis based on the degree of luminal narrowing and its geographical significance. The degree of stenosis and the location of the coronary artery lesion were scored as follows: 1 point for ≤25% stenosis, 2 points for 26–50% stenosis, 4 points for 51–75% stenosis, 8 points for 76–90% stenosis, 16 points for 91–99% stenosis, and 32 points for complete occlusion. Each lesion score was multiplied by a factor to take into account the importance of the lesion’s position in the coronary circulation: 5 for the proximal segment of the circumflex artery, 1.5 for the middle segment of the left anterior descending coronary artery, 1.0 for the right coronary artery, the distal segment of the left anterior descending coronary artery, the posterolateral artery, and the posterolateral artery, and 0.5 for the other segments. Finally, the Gensini score was calculated by summing the scores of the individual coronary segments [22]. All patients diagnosed with SAP underwent tests to assess coronary artery disease.

### 2.4. Patients’ Grouping

After CA or CCTA, or both, patients were divided into two groups: patients without coronary stenosis (patients without CAD group) and patients with any coronary stenosis (patients with CAD group). Most patients were diagnosed with CAD (87.95%, *n* = 146).

Patients were divided three ways: (1) according to CAD presence or absence; (2) according to Gensini score; (3) according to CAD-RADS score.

Patients who underwent CA were divided into three groups according to a Gensini score designed to assess the relationship between CAD and its severity. The first group consisted of patients with a Gensini score between 0 and 11 (*n* = 44), the second group consisted of patients with a Gensini score between 12 and 35 (*n* = 36), and the third group consisted of patients with a Gensini score above 35 (*n* = 27).

Six groups were used according to the CAD-RADS classification: CAD-RADS 0—no plaque or stenosis, CAD-RADS 1—minimal stenosis: 1 to 24%, CAD-RADS 2—mild stenosis: 25 to 49% and CAD-RADS 3—moderate stenosis: 50 to 69%, CAD-RADS 4—severe stenosis: 70 to 99% or left main ≥ 50% or three-vessel obstructive disease ≥ 70%, CAD-RADS 5—at least one completely occluded coronary artery [21].

Our patients who underwent CCTA were divided into four groups according to the CAD-RADS classification: the first group consisted of patients with no coronary stenosis (CAD-RADS 0; *n* = 13), the second group consisted of patients with minimal and mild stenosis (CAD-RADS 1 and 2; *n* = 26), the third group consisted of patients with moderate stenosis (CAD-RADS 3; *n* = 21), and finally, the fourth group consisted of patients with significant stenosis (CAD-RADS 4 and 5; *n* = 39).

### 2.5. Statistical Analysis

Statistical analysis was carried out using the program IBM-SPSS Statistics 29.0. Normality tests (Kolmogorov–Smirnov or Shapiro–Wilk) were used to test whether quantitative variables are normally distributed, given the sample size. Continuous variables are reported using means and standard deviation or medians (minimum and maximum values), depending on the distribution model. Categorical variables are presented as numbers and percentages.

Quantitative variables with normal and abnormal distributions were compared using the Student’s *t*-test and the Mann–Whitney U test. Categorical variables were compared using the chi-square test. The association between SII and the severity of CAD and other variables was assessed using Spearman’s rank correlation coefficient. The Kruskal–Wallis test was used to assess differences in variables between three or more groups. A two-sided *p* < 0.05 was considered statistically significant for all comparisons.

Receiver operating characteristic (ROC) curve analysis was performed to calculate the sensitivity and specificity of the estimated diagnostic parameters to predict the outcome (presence and severity of CAD). An area under the curve (AUC) of less than 0.5 was considered as non-diagnostic. The Youden index was used to determine the best cut-off value.

## 3. Results

### 3.1. Baseline Characteristics of the Patients

A total of 166 patients were investigated in this study. The majority of the patients underwent CA (71.69% (*n* = 119)) and more than half of all subjects underwent CCTA (59.64% (*n* = 99)). More than a quarter of patients (27.71% (*n* = 46)) had only one CCTA, while almost a third of patients (31.93% (*n* = 53)) had both tests to assess coronary atherosclerosis. More than a third of patients underwent CA (39.76% (*n* = 66)). The majority of patients had coronary atherosclerosis (87.95% (*n* = 146)). Less than half of these patients needed coronary revascularization: percutaneous coronary intervention was performed in 38% (*n* = 63), while 3% (*n* = 5) required coronary artery bypass grafting.

Patients diagnosed with CAD were statistically significantly older (*p =* 0.007) and had higher systolic blood pressure *(p* = 0.034) compared to patients without CAD (Table 1). Aspirin use was statistically significantly more common in patients with CAD *(p* = 0.047, respectively (Table 2).

A comparison of baseline characteristics and medication use of the patients ranked according to the Gensini score is shown in Table 3 and Table 4. No significant differences were found.

Baseline characteristics and drug usage in the groups according to the CAD-RADS classification are presented in Table 5 and Table 6. Patients with moderate (50–69%) or severe (more than 70%) coronary stenosis, or occlusion of at least one coronary artery, were statistically significantly older in comparison to patients without coronary stenosis (Group 1), (*p* ≤ 0.001). Patients with mild or minimal (1–49%) coronary stenosis were statistically significantly more likely to have arterial hypertension in comparison with patients without coronary stenosis (Group 1), (*p* = 0.002; *p* = 0.004; *p* = 0.023, respectively).

Statins were used more frequently in patients with the most severe coronary stenosis (Group 4, Table 6). The differences in usage of ACE inhibitors, beta-blockers, and aspirin are presented in Table 6. Usage of other drugs did not differ.

### 3.2. Lipidogram Readings

Total cholesterol, HDL-C, LDL-C, triglycerides, apoB, and Lp(a) levels did not differ between patients with and without CAD. Similarly, lipid metabolism readings did not differ between all Gensini score and patients without CAD and CAD-RADS groups.

### 3.3. Inflammation and Total Blood Count Readings

#### 3.3.1. Inflammation and Total Blood Count Readings in Patients Grouped According to CAD Presence

Platelet, erythrocyte count, lymphocyte count and percent, and LMR were significantly lower in patients with CAD *(p* = 0.019; *p* = 0.037; *p* = 0.032; *p* < 0.001; *p* < 0.001, respectively), while percentage and absolute neutrophils count and NLR were significantly higher in this group compared to patients without CAD *(p* = 0.002; *p* = 0.021; *p* < 0.001, respectively). Other markers of systemic inflammation, such as hs-CRP, MLR, SII, and SIRI, were statistically significantly higher in the CAD group (*p* = 0.048; *p* < 0.001; *p* = 0.018; *p* < 0.001, respectively), (Table 7 and Table 8).

**Figure 1:**
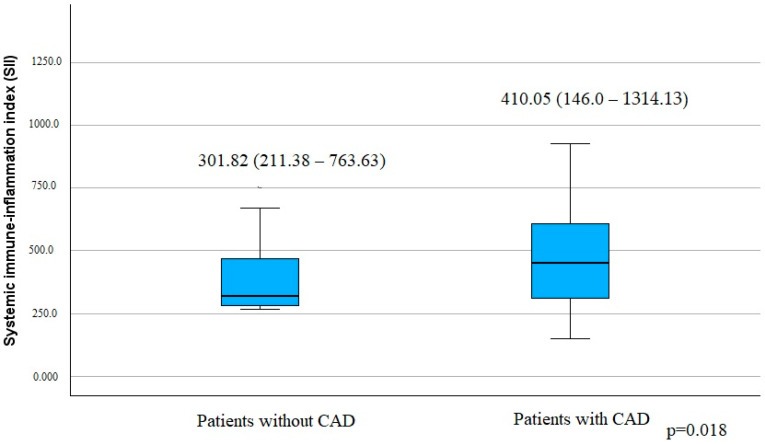
Systemic immune-inflammation index among the groups according to CAD presence. Values are medians, with minimum and maximum values in parentheses. CAD, coronary artery disease.

Patients with CAD had a statistically significantly higher SII compared to patients without the disease (Figure 1).

#### 3.3.2. Inflammation and Total Blood Count Readings in Patients Grouped by Gensini Score

Inflammation and total blood levels in patients grouped by Gensini score and in patients without CAD are shown in Table 9 and Table 10. Our results showed a tendency to increase in leukocyte count and percentage with the increase in the Gensini score group (with more severe coronary stenosis). We also found that the lymphocyte count and percent have a tendency to decrease with the increase of the Gensini score group. Monocyte count and percent did not differ between the Gensini score groups. The highest percentage of lymphocytes was found in the first Gensini group (with the least significant coronary artery disease) compared to the second and third Gensini groups (*p* < 0.001, for all).

The NLR, MLR, SII, PLR, and SIRI values were the highest in the third Gensini group (with the most severe coronary disease), and the LMR value was the lowest in the third group (with the most severe coronary disease), compared with the first Gensini group (with the least severe coronary disease) and the group without CAD (*p* < 0.001, for all).

**Figure 2:**
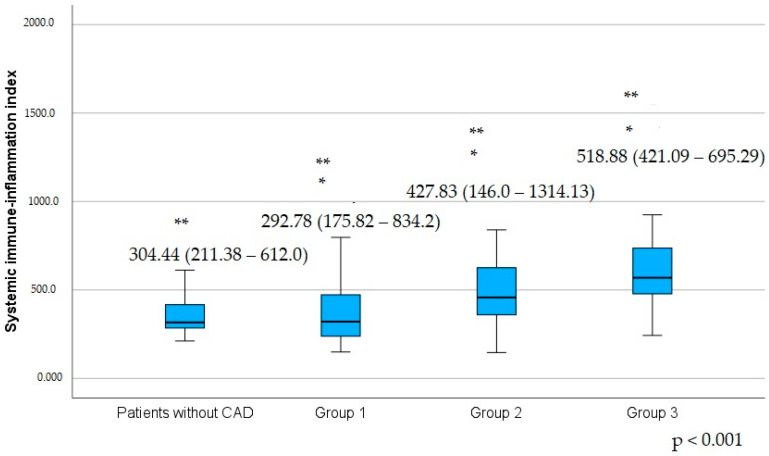
Difference of the systemic immune-inflammation index in the patients grouped according to Gensini score and patients without CAD. Values are medians, with minimum and maximum values in parentheses. CAD, coronary artery disease. Group 1: patients with a Gensini score between 0 and 11; Group 2: patients with a Gensini score between 12 and 35; Group 3: patients with a Gensini score above 35. The marker (*) indicates a statistically significant difference between the first Gensini group with the second and the third Gensini groups. The marker (**) indicates a statistically significant difference between the third Gensini group and the first and the second Gensini groups and patients without CAD.

Hs-CRP has the tendency to increase with the increase of Gensini score group (Table 9). It should be emphasized that the first Gensini group patients had a significantly lower SII, NLR, and SIRI than patients in the second and third Gensini groups (with the more severe CAD) (Table 10), (Figure 2).

#### 3.3.3. Inflammation and Total Blood Counts Readings in Patients Grouped According to CAD-RADS Classification

**Figure 3:**
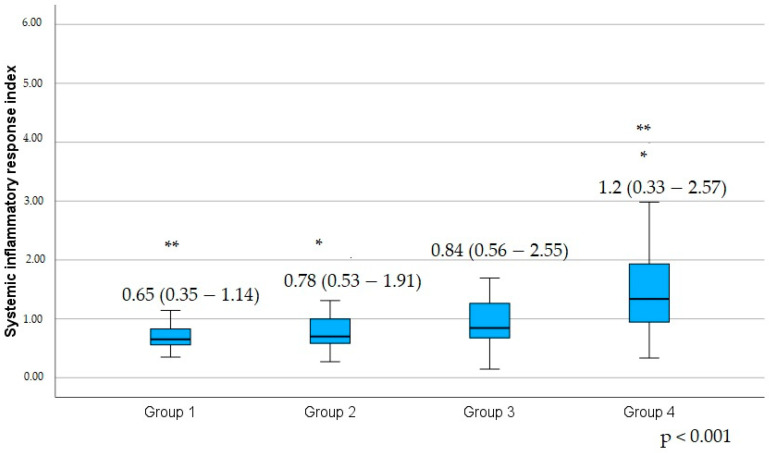
Difference of the systemic inflammatory response index in the patients grouped according to the CAD-RADS score. Values are medians, with minimum and maximum values in parentheses. Group 1 is patients with CAD-RADS 0—no plaque or stenosis; Group 2 is patients with CAD-RADS 1—minimal stenosis: 1 to 24% or CAD-RADS 2—mild stenosis: 25 to 49%; Group 3 is patients with CAD-RADS 3—moderate stenosis: 50 to 69%; Group 4 is patients with CAD-RADS 4—severe stenosis: 70 to 99%, or a left main ≥ 50%, or three-vessel obstructive disease ≥ 70% or CAD-RADS 5—at least one completely occluded coronary artery. The marker (*) indicates a statistically significant difference between the second group with the fourth CAD-RADS groups. The marker (**) indicates a statistically significant difference between the first and the fourth CAD-RADS groups.

Platelet and lymphocyte present had a tendency to decrease with increasing a number of CAD-RADS group (Table 11). Leukocyte count, neutrophil and monocyte count and percent, in contrast, had a tendency to increase. Patients without coronary stenosis (Group 1) had lower NLR, MLR, SIRI, and MHR compared to patients with the most severe coronary stenosis (Group 4) (*p* = 0.023; *p* < 0.001; *p* = 0.029; *p* = 0.007; *p* < 0.001; *p* < 0.001; *p* < 0.001; *p* < 0.001; and *p* = 0.010, respectively). Meanwhile, lymphocyte percentage and LMR were significantly higher in patients without coronary stenosis (Group 1) compared with patients with the most severe stenosis (Group 4), (*p* = 0.003; *p* < 0.001). LMR was also significantly higher in patients with minimal and mild coronary stenosis (Group 2) compared with patients with the most severe coronary stenosis (Group 4), (*p* = 0.046). Our results also showed that patients with the most severe coronary stenosis (Group 4) had significantly higher leucocyte, neutrophil, and monocyte counts, as well as SIRI and MHR, compared with patients with minimal and mild coronary stenosis (Group 2), (*p* = 0.002; *p* = 0.001; *p* < 0.001; *p* < 0.001; *p* < 0.001; *p* < 0.001; *p* = 0.036, respectively, Table 12 and Figure 3).

### 3.4. Correlations Between Laboratory, Echocardiography, and Baseline Characteristic Readings

#### 3.4.1. Correlations Between Echocardiography, Total Blood Count, and Inflammation Readings

Correlations between echocardiography and total blood count readings presented in Table 13. Accordingly, IVS and LVPW thickness weakly correlated with hs-CRP levels (*r* = 0.360, *p* < 0.001; *r* = 0.256; *p* = 0.021, respectively). There was a weak correlation between LVM and hs-CRP levels, SIRI, and NLR (*r* = 0.291, *p* = 0.008; *r* = 0.206, *p* = 0.01; *r* = 0.229, *p* = 0.004, respectively).

RWT weakly correlated with MLR (*r* = −0.228, *p* = 0.005) and LMR (*r* = 0.228, *p* = 0.005). LA volume weakly correlated with MLR (*r* = 0.217, *p* = 0.047) and LMR (*r* = −0.217, *p* = 0.047). LA volume index weakly correlated with NLR (*r* = 0.242, *p* = 0.038) and SIRI (*r* = 0.244, *p* = 0.036). NLR weakly correlated with e′_lat_ (*r* = −0.243, *p* = 0.016). No significant correlations were found between RV, RA, tricuspid annular systolic velocity, PASP, and total blood count and inflammation readings. Other correlations between echocardiography and calculated readings are presented in Table 14.

#### 3.4.2. Correlations Between Lipid, Total Blood Count, and Inflammation Readings

Correlations between total blood count, inflammation, and lipidogram readings showed some low statistically significant correlations (Table 15 and Table 16). Hs-CRP levels correlated with neutrophil percentage (*r* = 0.296, *p* = 0.007), neutrophil count (*r* = 0.298, *p* = 0.007), percentage of lymphocytes (*r* = −0.279, *p* = 0.011), value of NLR (*r* = 0.292, *p* = 0.008), NMR (*r* = 0.260, *p* = 0.018), and SIRI (*r* = 0.221, *p* = 0.046).

NMR very weakly correlated with HDL (*r* = 0.184, *p* = 0.028). No significant correlations were found between NLR, SII, and lipidogram readings.

PLR values weakly correlated with leukocyte counts (*r* = −0.362, *p* < 0.001) and NLR, NMR, and SII values (*r* = 0.284, *p* < 0.001; *r* = 0.256, *p* < 0.001; *r* = 0.295, *p* < 0.001, respectively) and moderately correlated with SIRI and MHR values (*r* = 0.532, *p* < 0.001; *r* = 0.542, *p* < 0.001, respectively).

There was a weak correlation between a higher platelet count and lymphocyte count (*r* = 0.220, *p* = 0.005) and percentage of monocytes (*r* = −0.263, *p* < 0.001), and a very weak correlation with monocyte count (*r* = −0.164, *p* = 0.035). Platelet counts weakly correlated with PLR, NMR, and LMR values (*r* = 0.398, *p* < 0.001; *r* = 0.208, *p* = 0.007; *r* = 0.301, *p* < 0.001, respectively) and with MLR (*r* = −0.301, *p* < 0.001) and MHR (*r* = −0.231, *p* = 0.005). SIRI correlated very weakly with platelet count (*r* = −0.170, *p* = 0.029). In contrast, MPV correlated very weakly with platelet count and SII (*r* = −0.197, *p* = 0.011; *r* = −0.165, *p* = 0.034, respectively) and weakly with PLR (*r* = −0.261, *p* < 0.001).

#### 3.4.3. Correlation Between Calculated Total Blood Count Readings and Severity of CAD

**Figure 4:**
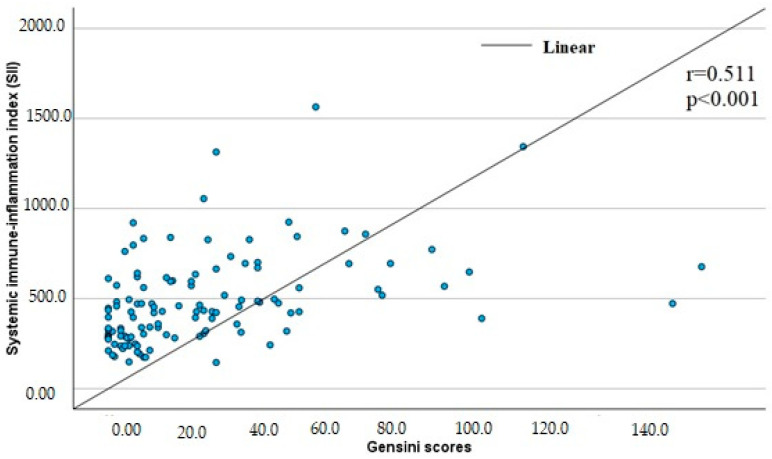
Correlation of SII with the degree of coronary artery disease according to the Gensini score. The severity of coronary artery stenosis as assessed by the Gensini score was found to be weakly correlated with PLR, NMR, MLR (*r* = 0.265, *p* = 0.004; *r* = 0.269, *p* = 0.003; *r* = 0.356, *p* < 0.001, respectively), and LMR (*r* = −0.355, *p* < 0.001), and moderately correlated with NLR and SIRI (*r* = 0.567, *p* < 0.001; *r* = 0.474, *p* < 0.001, respectively).

In terms of correlation with the severity of CAD, there was a positive mean correlation between SII and the severity of coronary stenosis according to the Gensini score (*r* = 0.511, *p* < 0.001) (Figure 4).

In addition, the CAD-RADS classification showed that the severity of coronary stenosis was weakly correlated with NLR, SII, and MHR (*r* = 0.365, *p* < 0.001; *r* = 0.239, *p* = 0.018; *r* = 0.346, *p* < 0.001, respectively) and moderately correlated with LMR (*r* = −0.454, *p* < 0.001), MLR, and SIRI (*r* = 0.455, *p* < 0.001; *r* = 0.522, *p* < 0.001, respectively). Clearly, there was a statistically significant strong positive correlation between CAD-RADS and Gensini score (*r* = 0.674, *p* < 0.001).

### 3.5. Correlations Related to Readings of Baseline Characteristics

#### 3.5.1. Correlations Between Baseline and Lipid Readings

Our study showed that smoking was weakly but statistically significantly associated with levels of triglycerides (*r* = 0.357, *p* = 0.003) and levels of HDL-C (*r* = −0.252, *p* = 0.034). BMI correlated with HDL-C levels (*r* = −0.299, *p* = 0.04). Older age correlated with dyslipidemia (*r* = 0.238, *p* = 0.026). Triglyceride levels weakly correlated with LVEDDi (*r* = −0.275, *p* = 0.033), e′_sep_ (*r* = −0.399, *p* = 0.014), and e′_lat_ (*r* = −0.341, *p* = 0.039). Uric acid levels correlated with HDL-C (*r* = −0.339, *p* = 0.030) and triglycerides levels (*r* = 0.336, *p* = 0.037).

#### 3.5.2. Correlation Between Baseline and Echocardiography Readings

Age correlated with LVEF (*r* = −0.239, *p* = 0.028) and PWT (*r* = −0.262, *p* = 0.021). Diastolic blood pressure correlated with PWT (*r* = 0.268, *p* = 0.018), LVM (*r* = 0.306, *p* = 0.007) and RV S’ (*r* = 0.302, *p* = 0.011). BMI correlated with LVEDD (*r* = 0.340, *p* = 0.003). Presence of AH correlated with IVS thickness (*r* = 0.242, *p* = 0.034), LVM (*r* = 0.316, *p* = 0.005) and LVMi (*r* = 0.331, *p* = 0.003). Smoking weakly correlated with LVEDDi (*r* = 0.357, *p* = 0.002). In addition, e′_sep_ moderately correlated with uric acid levels (*r* = −0.429, *p* = 0.036).

#### 3.5.3. Correlations Between Baseline and Total Blood Count and Inflammation Readings

Smoking correlated with uric acid concentration (*r* = 0.295, *p* = 0.044) and erythrocyte count (*r* = 0.371, *p* = 0.001). AH correlated with leukocyte, neutrophil, and monocyte counts (Table 17). Obesity correlated with uric acid levels (*r* = 0.299, *p* = 0.041). An early history of cardiovascular disease correlated with neutrophil percentage (*r* = 0.230, *p* = 0.036). Lymphocyte counts correlated with systolic and diastolic blood pressure (*r* = 0.223, *p* = 0.041; *r* = 0.280, *p* = 0.01, respectively).

Age weakly correlated with lower erythrocyte count (*r* = −0.324, *p* = 0.003), lymphocyte count (*r* = −0.291, *p* = 0.034), and diastolic BP (*r* = −0.365, *p* < 0.001). Some weak correlations were found between calculated total blood count and baseline readings (Table 17).

### 3.6. Correlations Related to Drug Usage

Some weak correlations have been found between the readings of lipid concentration, total blood counts and drug use (Table 18 and Table 19). As shown in Table 19, aspirin has a positive weak association with NLR, MLR, SII, and SIRI, and a negative association with LMR; the same association was found with statin use (except for SII). Thus, it can be concluded that the use of medications can potentially influence the inflammatory parameters assessed.

Additional correlations were found: the use of beta-blockers, ACEi, or ARB and statin was associated with age (*r* = 0.301, *p* < 0.001; *r* = 0.374, *p* < 0.001; *r* = 0.198, *p* = 0.010, respectively), and leukocyte counts correlated with statin (*r* = 0.157, *p* = 0.044) and aspirin (*r* = 0.223, *p* = 0.004) use, while platelet counts correlated negatively with statin use (*r* = −0.211, *p* = 0.007). There were no significant associations between aspirin and CaCB and lipid and uric acid readings. Total blood readings were not associated with CaCB and MRA.

### 3.7. ROC Curve Analysis to Determine the CAD Severity of the Estimated Parameters

**Figure 5:**
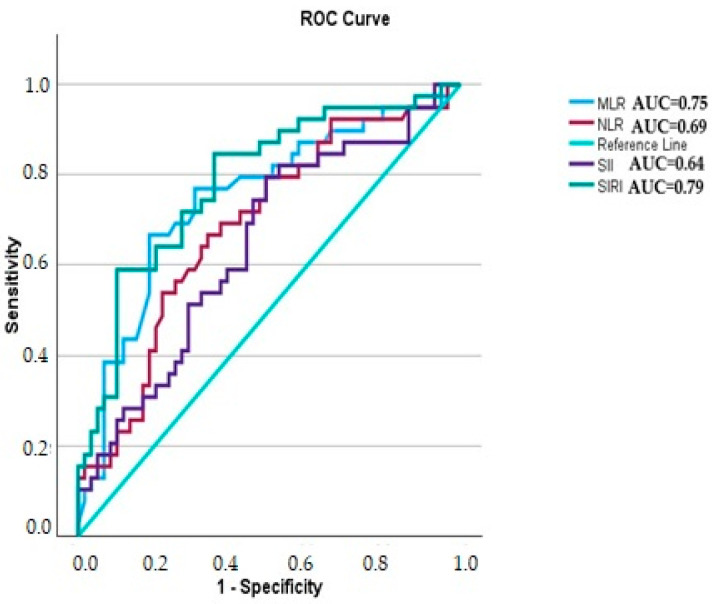
ROC curve showing the usefulness of the estimated total blood parameters in predicting the most severe CAD (as assessed by the CAD-RADS score). NLR, neutrophil-to-lymphocyte ratio; MLR, monocyte-to-lymphocyte ratio; SII, systemic immune-inflammation index; SIRI, systemic inflammation response index; AUC, area under the ROC curve.

ROC curve analysis showed that calculated parameters, such as MLR, NLR, SII, and SIRI, were useful in identifying the most severe coronary stenosis as assessed by the CAD-RADS score (Group 4). This is illustrated in Figure 5.

SIRI optimal cut-off value was 0.88 (*p* = 0.0001). At this cut-off value, the sensitivity was 85% and the specificity 64%. In addition, the MLR had a cut-off value of 0.31 (*p* = 0.0001) with a sensitivity of 67% and a specificity of 81%. The cut-off value of NLR was 1.87 (*p* = 0.001) and balanced 69% sensitivity and 63% specificity. SII had a cut-off value of 387.33 (*p* = 0.012) with a sensitivity of 80% and a specificity of 51%.

**Figure 6:**
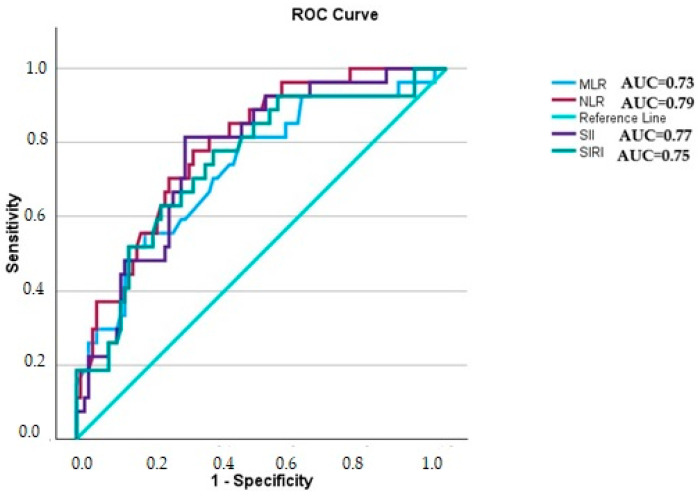
ROC curve showing usefulness of estimated total blood parameters in predicting the most severe CAD (as assessed by the Gensini score). NLR, neutrophil-to-lymphocyte ratio; MLR, monocyte-to-lymphocyte ratio; SII, systemic immune-inflammation index; SIRI, systemic inflammation response index; AUC, area under the ROC curve.

ROC curve analysis showed that NLR, SII, SIRI, and MLR were also useful in detecting the most severe coronary stenosis (Group 3) and the severity of CAD, as assessed by the Gensini score, in patients who underwent CA. This is shown in Figure 6. The maximum NLR optimal cut-off value was 2.16 (*p* = 0.000) with the sensitivity of 78% and the specificity of 69%. SII had a cut-off value of 472.8 (*p* = 0.000), with a sensitivity of 82% and a specificity of 70%. The cut-off value for SIRI was 1.45 (*p* = 0.000) with a balanced sensitivity of 63% and specificity of 77%. MLR had a cut-off value of 0.4 (*p* = 0.000), with a sensitivity of 52% and a specificity of 86%.

## 4. Discussion

This pilot retrospective study of patients with SAP shows that an easily measurable hematological markers, such as SII, SIRI, NLR, MHR, MLR, and LMR could indicate the severity of atherosclerosis. LMR and SIRI could be the best readings for chronic inflammation evaluation in the SAP patients. SIRI could reflect the degree of atherosclerosis best of all other calculated total blood count readings.

### 4.1. Differences in Laboratory Parameters (Calculated Total Blood Counts Readings, Lipids and hs-CRP) Between the Patients’ Groups

Our study showed that patients with CAD had higher NLR, SIRI, MLR, and SII and lower LMR in comparison to patients without CAD. This can be explained by pathogenetic mechanisms, which have been described in detail in some publications.

For instance, neutrophils secrete neutrophil extracellular traps, pro-inflammatory and pro-thrombotic substances that trap leukocytes and promote thrombosis [8,9,23]. In patients with severe CAD, neutrophil counts are often elevated, primarily due to acute and chronic inflammatory reactions, tissue repair mechanisms, and stress-induced mobilization [9]. In addition, neutrophils can infiltrate endothelial tissue and release pro-oxidants and pro-inflammatory mediators, which in turn can form neutrophil extracellular traps and promote the formation and development of atherosclerotic plaques [24]. It should be mentioned that recent studies have shown that neutrophils play an important role in the production of reactive oxygen species and the environment during thrombus formation [17,25]. In our patients, neutrophil count was significantly higher in the CAD group (Table 7) and increased with increasing in Gensini (Table 9) and CAD-RADS score number (Table 11). Reduced lymphocyte numbers could be associated with the progression of atherosclerosis [8,9,26]. Our results show lower lymphocyte counts in patients with CAD (Table 7), gradually decreasing with increasing degree of atherosclerosis (Table 9 and Table 11). The combination of neutrophil and lymphocyte parameters is presented as a better prognostic value than either of them alone [23,26]. In our study, NLR was found higher in the CAD group (Table 8) and increased in Gensini (Table 10) and CAD-RADS groups as well (Table 12). Even more, the results of a study conducted on NLR in patients with CAD recently showed that increased NLR was a predictor of CAD prognosis (by a factor of 2.79) [27]. Thus, our study was in consensus with these results, as we also found that patients with CAD had a significantly higher NLR compared with patients without CAD. In addition, it seems NLR could be a useful marker for evaluating the severity of CAD.

Some studies have shown that high monocyte and low lymphocyte counts are independent predictors of CAD risk, and the MLR index integrates the risk of coronary lesion severity [28,29,30,31]. As mentioned earlier, it has been hypothesized that monocytes may enter the arterial wall, differentiate into macrophages, and activate the secretion of pro-inflammatory cytokines, the production of matrix metalloproteinases, and the production of reactive oxidative species, which are key players in the initiation of atherosclerotic plaque initiation and the formation or rupture of atherosclerotic plaques [8,9,29]. Lymphocytes are potentially important immune cells in cardiovascular disease, and low lymphocyte numbers have been shown to reflect impaired coronary microcirculation, which has been confirmed as an important mechanism in the pathogenesis of CAD [8,9,30]. We found that MLR was significantly higher in patients with CAD (Table 8). Ji H. et al. reported similar results in their study and confirmed that MLR may be a risk factor for atherosclerosis and correlate with CAD severity. Their results showed that MLR > 0.18 predicted CAD with a sensitivity of 69.03% and specificity of 64.81% [31]. It should be mentioned that no difference in monocyte count was found between the groups according to CAD presence (Table 7) and Gensini score (Table 9). But, monocyte count increases gradually when CAD-RADS group number increased (Table 11). Our results show MLR could give more information related to patients‘ condition related to atherosclerosis as cell count.

Kose N. et al. attempted to assess the association between LMR and CAD in patients with stable angina pectoris in their study. Their exclusion criteria were similar to ours. The results of their study showed that LMR was an independent predictor of CAD severity in patients with stable CAD. Their results showed that patients with CAD had lower LMR values (4.5 ± 3.2 vs. 6 ± 2.9, *p* < 0.001) compared to the group without CAD [32]. In our study, the same trend of LMR values was also observed in these groups (Table 8). Recent studies show that LMR can also be used as a marker of inflammation, just like the other markers mentioned above (NLR and MLR). LMR combines two independent markers of inflammation, and both high monocyte counts and low lymphocyte counts are associated with coronary atherosclerosis [8,9,29,30,33]. Monocytes are recruited in the intima and subintima, differentiate into macrophages and mast cells in response to a number of locally produced cytokines, and initiate the atherosclerotic process by supporting plaque formation [34]. Meanwhile, lymphocytes play an important role in the pathogenesis of atherosclerosis by modulating the immune response, with different subtypes promoting or inhibiting plaque growth and stability. Lymphocytes, especially T cells, play a key role in modulating the immune response in atherosclerotic plaques. Regulatory T cells (Tregs) have been shown to have anti-inflammatory properties and attenuate atherosclerosis by reducing inflammation in atherosclerotic plaques [35]. The combined role of lymphocytes and monocytes in atherosclerosis highlights the chronic inflammatory process of the disease and may lead to the progression of atherosclerosis [33,34,35]. Thus, our discovery of decreased LMR and increased MLR in patients with CAD and decreasing LMR and increasing MLR with increasing Gensini score and CAD-RADS group number supplements recent research findings.

In addition, other indicators calculated from a complete blood count may be relevant to the state of inflammation and the pathogenesis of atherosclerosis as well. Urbanowicz T. and co-authors attempted to assess the predictive role of SIRI for CAD in their retrospective study. Their study included 256 patients, and excluded patients with acute coronary syndromes or comorbid hematological, rheumatic, and oncological diseases. The results of their study were similar to our study and confirmed the trend that SIRI was the highest in patients with more severe CAD (complex (2- or 3-vessel) coronary disease). For example, in their study, the median SIRI was the highest in the group of patients with complex CAD (0.99 (0.76–1.27)), compared with the group without CAD (0.82 (0.57–1.06), *p* < 0.001). In addition, a logistic regression analysis performed in their study in patients with single-vessel CAD compared to patients without coronary disease showed that SIRI can be a predictive laboratory parameter (OR: 3.32, 95% CI: 1.56–7.03, *p* = 0.002) [16]. As well, our results showed that patients who underwent CA with CAD severity assessed by the Gensini score also had the highest SIRI in the group, with the most severe CAD (Group 3, Table 10). The same trend was observed in our patients who underwent CCTA and had their coronary disease severity assessed by the CAD-RADS score (Group 4, Table 12). It seems that elevated SIRI levels, which indicate an imbalance between high neutrophil and monocyte counts and low lymphocyte counts, could be indicative of an increased pro-inflammatory state. A high SIRI value could be a strong indication of increased risk of atherosclerosis, as it indicates an overactive immune response (involving even three types of leukocytes) that contributes to the formation and progression of atherosclerotic plaques. It is thought that low SIRI values may indicate a more balanced immune response and better regulation of inflammation, which may protect against atherosclerosis [16,36].

Moreover, this parameter is useful in predicting long-term outcome and mortality risk; for example, Urbanowicz T. and colleagues found in their study that SIRI may be a predictor of poorer long-term outcome after off-pump coronary artery bypass grafting in patients with CAD. The results of their study showed that if the preoperative SIRI value exceeds 1.27, patients are at risk of mortality [37].

Two years ago, Ye Z. et al. published a systematic review supporting the idea that SII may be another prognostic marker of atherosclerosis, which, compared to traditional markers of inflammation, may be a valuable tool for predicting CAD risk, as it reflects the balance between inflammation and immunity, which is of great importance in the CAD context. High SII may be associated with increased inflammatory activity due to high neutrophil and platelet counts and low lymphocyte concentrations [9]. Scientists are still trying to find the answer to why SII increases in CAD. It is thought that in CAD, platelets are activated and accumulate at sites of vascular damage, contributing to thrombosis and subsequent inflammation. Thus, increased platelet activity is associated with systemic inflammation and may lead to higher SII values [19]. Additionally, leukocytes and monocytes, recruited by platelets, migrate to the site of inflammation and release inflammatory mediators such as chemokines and cytokines, which can trigger vascular inflammation. As already mentioned, monocytes play a key role in this inflammatory process by migrating to the site of atherosclerotic lesions, where they differentiate into macrophages and contribute to plaque formation. Thus, patients with severe CAD usually have elevated monocyte counts, mainly due to chronic inflammation, endothelial dysfunction, and elevated cytokine levels [19]. On the other hand, the decrease in lymphocyte counts in patients with severe CAD is primarily the result of chronic inflammation, dysregulation of the immune system, acute stress response, and possible migration of lymphocytes to inflamed tissues. In addition, the pro-inflammatory environment often leads to apoptosis of lymphocytes, resulting in a reduction in their number [24]. Our data showed that SII was higher in the CAD group in comparison with group without CAD. Accordingly, SII gradually increased with increasing in Gensini score group. But, it does not differ between the CAD-RADS score groups.

Hs-CRP is a valuable inflammatory factor in coronary disease as it is a marker of systemic inflammation, which plays an important role in the pathophysiology of atherosclerosis. Elevated levels of hs-CRP reflect a state of inflammation that can be related with endothelial dysfunction, plaque formation, and increased plaque instability and vulnerability [38]. This is the reason why we chose to evaluate hs-CRP in our study.

Behera D. et al. confirmed the importance of hs-CRP as a significant risk factor for CAD. The results showed that hs-CRP levels were significantly higher in the group of patients with CAD compared with patients without CAD (2.932 ± 0.605 vs. 0.379 ± 0.202 mg/dL, *p* < 0.001) [39]. Their results were similar to ours, and we also found significantly higher hs-CRP values in the CAD group compared to the group without CAD (Table 6).

Cederström S. et al. recently analyzed the relationship between hs-CRP and the degree of atherosclerosis in CAD. Their population cohort was large, comprising 25,408 patients, but the results showed that elevated hs-CRP may be only weakly associated with the presence of coronary atherosclerosis [40]. Also, we found no significant changes in hs-CRP levels when assessing the severity of CAD. Similar results were reported by Bouzidi N et al. in their study, who also concluded that hs-CRP levels were not associated with the severity of CAD, as assessed by the degree of stenosis and the number of coronary vessels affected [41].

We found that lipid concentrations did not differ between the investigated groups. Other research work similarly found no statistically significant differences between total cholesterol, triglycerides, LDL-C, and uric acid levels in the two groups of patients with and without CAD [42]. We think the absence of differences could be related to statin usage.

In conclusion it could be stated that our results showed NLR, MLR, SIRI, and SII were higher and LMR was lower in the CAD group. NLR, MLR, SIRI, and SII increased and LMR decreased gradually with an increase of the Gensini score group. There was a tendency MHR and SIRI to increase and LMR to decrease when the group number of CAD-RADS increased. SII did not differ between the CAD-RADS groups. LMR and SIRI increased gradually with an increase of the Gensini score group. Thus, LMR and SIRI could be the best readings for chronic inflammation evaluation in patients with SAP, especially when hs-CRP does not differ between the investigated groups according to Gensini and CAD-RADS score.

### 4.2. Discussion of Correlations

#### 4.2.1. Correlation Between Readings Based on Total Blood Count Data and Lipids and hs-CRP

Our study showed that lipidogram reading HDL-C level reversibly correlated with leukocyte, neutrophil, and monocyte counts, percentage of monocytes, and MLR, SIRI, and MHR, while HDL-C levels were only weakly correlated with PLR and LMR. But, neither triglyceride nor HDL-C differed between the patients grouped according to CAD presence, Gensini score, and CAD-RADS classification in our study (Table 7, Table 8 and Table 9). Thus, it seems only HDL-C concentration could be related with leukocyte, monocyte, and neutrophil counts in SAP patients.

It is known that elevated lipid levels can lead to increased involvement and activation of immune cells, which promote the progression of CAD and associated with cardiovascular risk. When lipids, especially oxidized LDL, accumulate in the intima of the artery, they activate the immune system, leading to an increase in the production of leucocytes and the recruitment of monocytes and neutrophils to the site of inflammation. Lipid concentration is presented as closely linked to the number of leukocytes, monocytes, and neutrophils through a variety of mechanisms related to inflammation, immune activation, and atherosclerotic processes [43]. One study showed that levels of HDL-C associated reversibly with MLR and SIRI, mainly due to HDL-C’s anti-inflammatory properties. In addition, HDL-C may inhibit the expression of endothelial cell adhesion molecules, thereby reducing the recruitment of immune cells such as monocytes and lymphocytes to inflamed tissues. Higher levels of HDL-C may stimulate the differentiation of T regulatory cells, which may help to suppress inflammatory reactions and reduce lymphocyte activation, thus contributing to lower MLR. In addition, the ability of HDL-C to transport cholesterol, reduce inflammation, improve endothelial function, and modulate immune responses contributes to the inverse relationship between HDL-C levels and SIRI [44]. Our findings are in consensus with these findings. In our study, HDL-C reversibly correlated with MLR and SIRI and positively correlated with LMR, despite the fact that lipid concentration did not differ between the groups analyzed.

As already mentioned, monocytes are involved in atherosclerosis by migrating into the subendothelial space and taking up lipoproteins. In contrast, HDL-C has anti-atherosclerotic properties and HDL-C molecules prevent monocyte activation and recruitment [15]. Thus, it can be assumed that higher HDL-C levels (reflected by lower MHR) reflect an increased cholesterol efflux capacity, leading to reduced lipid accumulation in macrophages and reduced mast cell formation, as found in our study. We found that MHR correlated with all the lipid readings (total cholesterol, LDL, HDL, and Lp(a) concentrations) except apoB (Table 15). Thus, our results supplement findings about the relationship between lipid levels and inflammatory statement in the atherosclerosis by possibly modulating inflammatory reactions.

Our study showed that hs-CRP levels positively correlated with neutrophil counts and percentage, NLR, NMR, and SIRI, and reversibly correlated with percentage of lymphocytes. He J. et co-authors also found that NLR levels were modestly and significantly correlated with hs-CRP levels. This study demonstrated discordantly elevated NLR levels were associated with a greater risk of adverse clinical events in patients with stable CAD [45]. Bouzidi N. et al. in their study also found that inflammatory parameters of white blood cells were significantly increased in the patients with high hs-CRP levels, as was shown in our results as well. In addition, increased hs-CRP levels often reflect ongoing inflammation, and neutrophils are one of the first immune cells to respond to inflammation [41]. Thus, NLR, NMR, and SIRI (readings that involve neutrophil count) correlations with hs-CRP are logical, and could confirm low inflammation in SAP patients.

Concluding results about the correlations between readings based on total blood count data and lipids and hs-CRP, it could be stated that lipid concentration in the blood are related with leukocyte, monocyte, lymphocyte, and neutrophil counts in SAP patients. Hs-CRP correlation with NLR, NMR, and SIRI confirm that NLR, NMR, and SIRI show the inflammation in these patients.

#### 4.2.2. Correlation Between Echocardiography Readings and Other Estimated Readings

Looking for correlation with cardiac echocardiography readings and laboratory parameters, we found that LVEF positively correlated with lymphocyte percentage and LMR, but negatively with the count and percentage of neutrophil, NLR, and MLR. In addition, LVEDD correlated with erythrocyte count, neutrophil count and percentage, NLR, SIRI, and MHR, and reversibly with lymphocyte percentage, platelet count, and LMR. LA diameter also negatively correlated with platelet count, lymphocyte percentage, and LMR, and positively with MLR, SIRI, and MHR. Hs-CRP directly correlated with IVS and LVPW thickness, and LVM. Meanwhile, Medenwald D. and co-authors also found that hs-CRP, can be associated with structural changes in the heart, in particular the mass of the left ventricle and the thickness of the interventricular septum and the left ventricular posterior wall [46]. Previous studies have shown that markers of inflammation such as hs-CRB, high leukocyte counts and NLR values are associated with left ventricular diastolic dysfunction [4,47]. Similarly, our study demonstrated that left ventricular diastolic function was correlated reversibly with count and percentage of lymphocytes, as well as LMR, and positively with count of neutrophil, NLR, MLR, and SIRI (Table 13 and Table 14). These relationships show the associations between the cardiac function and total blood count related inflammation readings.

#### 4.2.3. Correlation Between Readings Based on Total Blood Count Data and Severity of CAD

Several studies have looked at the relationship between calculated total blood count readings and the severity of coronary atherosclerosis (Table 16). Our study is unique in that we analyzed all the possible readings on the basis of total blood count, compared to other published studies. According to our knowledge, we first evaluated calculated total blood count readings’ relationship with the degree of atherosclerosis in SAP patients without comorbidities.

A growing number of studies have examined the role of SII in atherosclerosis and cardiovascular disease (CVD). In some research works, it was shown that elevated SII levels associate with an increased risk of CAD and predict more severe CAD [3,9]. Dai et al. estimated that elevated levels of SII significantly associate with the progression of atherosclerosis, which leads to slow coronary flow phenomenon, and associate with a higher number of diseased coronary vessels [19]. The recent meta-analysis mentioned above also showed that elevated SII levels are associated with an increased risk of CVD. In addition, some studies have hypothesized that systemic immune-inflammation plays a key role in destabilizing atherosclerotic plaques, leading to plaque rupture and subsequent development of acute coronary syndrome. Liu et al. also found a positive correlation between SII and the severity of coronary artery disease. As in our study, they also showed that a higher SII correlated with more severe coronary atherosclerosis assessed by the Gensini score [42]. Gur et al. showed in their study that elevated SII was significantly associated with greater myocardial damage in acute coronary syndrome (ACS) [48]. In contrast to this study, in our study we excluded patients with ACS and patients with diabetes in order to have more homogeneous groups and to eliminate the effects of other conditions on the immune and inflammatory systems. Candemir M et al. showed that a SII value above 750 could predict severe CAD with 86.2% sensitivity and 87.3% specificity [3]. However, it is important to note that, although the results are encouraging, the level of evidence for the use of SII as a CAD biomarker remains generally low [9]. This could be due to a number of factors, including differences in study designs, patient cohorts, and outcome measures across studies. Our results suggest that higher SII may reflect increased systemic inflammation, which in turn contributes to CAD progression. In our study, the SII positively correlated with Gensini (*r* = 0.511, *p* < 0.001) and CAD-RADS scores (*r* = 0.239, *p* = 0.018) (CAD severity), and could have a better predictive power for CAD onset than lipid fractions and hs-CRP.

Some authors looked for the other total blood count calculated readings. Candemir M et al. studied patients with SAP to assess the prediction of CAD severity by prognostic indicators derived from SII, NLR, PLR, and MLR. Their results show that NLR, PLR, MLR, and SII statistically significantly correlated with CAD severity, but SII was suggested as a risk factor for atherosclerosis and as better predictor of CAD severity than other total blood count calculated readings such as NLR, MLR, and PLR. [3].

Guo W. et al. found that patients with CAD had a higher NMR, and the increase in NMR was significantly associated with the severity of coronary atherosclerosis. The investigators of this study concluded that NMR may help to predict cardiovascular events in patients with CAD, and may be associated with vulnerable plaque burden and a higher risk of adverse cardiovascular events [14]. Our results also showed that NMR was statistically significantly higher in patients with more severe CAD compared with patients without CAD, and the NMR value was significantly higher according to the Gensini score (Groups 2 and 3, Table 10). However, we found no significant differences and no correlation between NMR and CAD-RADS score. This could be explained by the comorbidities of patients in Guo W et al. groups.

Interesting results from a recent study showed that the number of inflammatory cells can vary depending on the location of significant calcified coronary lesions. The relationship between neutrophil and lymphocyte counts in the blood was found to be positive, and the location of calcified atherosclerotic plaques may be indicative of the inflammatory background of epicardial atheroma formation and distribution [49]. The investigators highlighted that total neutrophil and lymphocyte counts were the best predictors of proximal localization, with a sensitivity of 79.07% and a specificity of 73.68% (AUC 0.747 (*p* < 0.001)).

Also, Liu Y. confirmed in his study that NLR can predict atherosclerosis with greater power, and NLR and PLR values positively correlated with atherosclerosis severity and Gensini score [42]. Other previous studies have also shown that NLR, PLR, and MLR can predict the inflammatory role of different cell types in atherosclerosis and may be predictors of coronary morbidity and mortality in patients with CVD [5,6,50]. In addition, Núñez J. et al. showed in their study that lower lymphocyte counts were associated with faster progression of atherosclerosis in coronary disease [51]. Meanwhile, Kiris T. et al. reported in their study that high MLR values were associated with vulnerable plaques in patients with stable angina [11]. These results also are in consensus with those of our study.

We found that NLR, MLR, SIRI, and SII were significant higher (Table 11, Figure 1) in patients with CAD. Furthermore, NLR, MLR, SII, SIRI, and MHR were the highest, and LMR the lowest in Group 3 of patients grouped by Gensini score (Table 13). Accordingly, neutrophil percentage was the lowest and lymphocyte percentage was the highest in the first group of patients grouped by Gensini score (Table 12). Therefore, our results supplement knowledge that neutrophil, lymphocyte, and monocyte count and calculated total blood count readings NLR, MLR, LMR, SIRI, SII, and MHR could be related with degree of atherosclerosis.

Last year, Chinese researchers Zhao et al. enrolled nearly 300 patients with suspected CAD confirmed by coronary angiography to assess prognostic indicators of systemic inflammation and factors related to lipid metabolism. Their study showed that MHR, NLR, MLR, NLR, and SII levels were significantly higher in CAD patients than in non-CAD patients, and the results showed that an MHR value of more than 0.47 and an SII value of more than 589.12 were considered as independent CAD risk factors [15].

Yildiz et al. showed that the SII can help to assess not only the severity but also the prognosis of CAD. Higher values of the SII predicted a higher 1-year rate of major adverse cardiac events [52]. The SII reported to be a more accurate predictor of inflammatory activity compared to a single hematological parameter. The SII may serve as a biomarker to complement the prognostic information that traditional risk factors cannot provide [9,52] (Table 20).

Several years ago, Chinese researchers Si Y. and colleagues organized a study to assess the relationship between LMR and coronary plaque burden associated with CAD. Their study population consisted of patients with suspected CAD with SAP symptoms. Exclusion criteria were acute coronary syndrome, renal failure, connective tissue diseases, severe valvular heart disease and hypertrophic cardiomyopathy, and pregnancy, but diabetics were included. Coronary computer tomographic angiography was used to confirm coronary artery stenosis and coronary artery calcification (CAC). The results of this study showed that patients with CAD tended to have a lower LMR value than patients without CAD (*p* = 0.001). Moreover, LMR was negatively correlated with CAC score and was an independent risk factor for CAC score (*p* < 0.05). The diagnostic cutoff point of LMR, calculated by multiple logistic regression model, showed that LMR ≤ 4.8 was a new independent risk factor for CAD (*p* < 0.05) [13]. Our results are in line with Si Y.’s findings: we found that LMR negatively correlated with coronary stenosis (CAD-RADS class number). Thus, it can be argued that low LMR, low lymphocyte counts, or high monocyte counts may induce inflammation and oxidative stress, releasing more pro-inflammatory factors, damaging the endothelium and suppressing the immune response, which in turn promotes the formation of mast cells and the deposition of sub-endothelial lipids [12]. We found MLR, NLR, and SII higher in CAD patients in comparison with without CAD as well (Table 11). Accordingly, SIRI was higher, and LMR was lower in our CAD patients’ group. It should be noted that Si Y. et al. also excluded patients with acute coronary syndrome, renal failure, connective tissue disease, severe valvular heart disease, and hypertrophic cardiomyopathy, but included patients with diabetes mellitus and ischemic stroke, and that these comorbidities may have had an impact on the calculated and tested inflammatory and total blood count readings [12].

In conclusion, we can state that LMR, NLR, MLR, MHR, SIRI, and SII are available biomarkers that could help assess the severity of coronary atherosclerosis in patients with SAP. Our study showed that NLR, SII, LMR, MLR, and SIRI have strongest correlations with CAD severity. However, further research works is needed to determine the clinical relevance of SIRI, LMR, MLR, and others in the evaluation of chronic inflammation in CAD, to confirm its usefulness in clinical practice, to establish standard cut-off values, and to elucidate the underlying mechanisms linking the readings to the pathophysiology of CAD. According to our knowledge, we first evaluated correlation between all total blood count calculated readings related to chronic inflammation and severity of CAD in the same patient’s population. It should be emphasized that our investigated patients did not have comorbidities, so the readings were not affected by other pathologies. Our findings confirm that SIRI and LMR could reflect the low chronic inflammatory statement in SAP patients best of all other readings, and they are most related to the degree of atherosclerosis in SAP patients. SIRI reflects the degree of atherosclerosis best of all other calculated total blood count readings.

### 4.3. Limitations

This study has several limitations. It is a pilot retrospective study evaluating the calculated total blood count readings related to low chronic inflammation as related with CAD severity in one small center. Therefore, the results of the association between the severity of coronary artery stenosis and the readings level should be further tested in studies with larger sample sizes. Another limitation is that plaque composition was not assessed in this study (no intravascular ultrasound or optical coherence tomography was used, nor was plaque composition assessed by CCTA), as the type of plaque may also influence calculated total blood inflammation-related readings. Coronary artery calcium score was also not assessed.

Another limitation of our study is that as it is retrospective and may have some selection bias, where the participants included in this study may not fully represent the overall target population of interest. However, we note that although our study is based on a retrospective design, we have used clear and predefined inclusion and exclusion criteria to ensure consistency. In addition, our study did not include patients with acute coronary syndrome (ACS), so the results may not be applicable to the whole spectrum of CAD, as these patients represent a significant subgroup of CAD patients. This was done because the inclusion of these patients may have led to erroneous results, because ACS is characterized by acute inflammation, endothelial dysfunction, and plaque rupture, which may temporarily alter the parameters we estimated and may not reflect the chronic inflammatory processes often seen in stable coronary artery disease (CAD), thus leading to misleading results. Accordingly, because the study was retrospective, we cannot measure the more specific markers for understanding immune function and atherosclerosis development, like VCAM-1 or CD93.

Finally, this was a short-term retrospective pilot study, so future adverse major cardiac events and mortality were not assessed.

## 5. Conclusions

NLR, MLR, SIRI, and SII were higher and LMR was lower in patients with CAD. LMR decreased and NLR, MLR, SIRI, and SII increased gradually with an increase in Gensini group number. LMR decreased and SIRI with MHR increased gradually with an increase in CAD-RADS group number. Thus, NLR, LMR, and SIRI seems to be the best indicators for chronic inflammation evaluation in patients with SAP.

Moderate correlation was found between inflammatory indexes SII, NLR, and SIRI and severity of coronary stenosis according to Gensini score. The CAD-RADS score moderately correlated with LMR, MLR, and SIRI. NLR, MLR, and SIRI correlated with some exocardiography left ventricle parameters. Thus, SIRI reflects the degree of atherosclerosis best of all. Accordingly, ROC curve analysis showed that SIRI could be most useful in identifying the most severe coronary stenosis as assessed by the CAD-RADS: SIRI optimal cut-off value was 0.88 (*p* = 0.0001, sensitivity—85% and specificity—64%). In detecting the most severe coronary stenosis (Group 3) and the severity of CAD, as assessed by the Gensini score, in patients who underwent CA, the cut-off value for SIRI was 1.45 (*p* = 0.0001) with a sensitivity of 63% and specificity of 77%. This value is easily measurable, and its low-cost ability to integrate multiple immune cells could make it a valuable marker for assessing systemic inflammation and atherosclerosis progression and degree in SAP patients.

## Figures and Tables

**Table 1:** Baseline characteristics of patient groups according to CAD presence.

	Patients with CAD (*n* = 146)	Patients Without CAD (*n* = 20)	*p*-Value
Age, years	60.5 (38–85)	53 (34–79)	0.007
Female gender, *n* (%)	60 (41.1)	10 (50)	0.477
BMI, kg/m^2^	29.18 (19.57–41.03)	28.55 (22.04–38.06)	0.240
Smoking, *n* (%)	43 (29.7)	5 (25)	0.796
Obesity, *n* (%)	59 (41.5)	6 (30)	0.610
Arterial hypertension, *n* (%)	125 (85.6)	15 (75)	0.495
Early family history of cardiovascular disease, *n* (%)	25 (19.4)	1 (5)	0.201
Systolic BP, mmHg	140 (116–185)	133 (100–180)	0.034
Diastolic BP, mmHg	88 (64–120)	83.5 (70–110)	0.582
Heart rate, bpm	68 (50–100)	70 (56–83)	0.496
Uric acid, μmol	334.5 (154–627)	345 (180–491)	0.862

Values are medians, with minimum and maximum values in parentheses or numbers with percentages in parentheses. CAD, coronary artery disease; BMI, body mass index; BP, blood pressure; bpm, beats per minute. Dyslipidemia group consisted of patients with total cholesterol concentration greater than 5 mmol/L or LDL-C concentration greater than 3 mmol/L or triglycerides concentrations greater than 1.7 mmol/L or HDL-C less than 1 mmol/L for men, <1.2 mmol/L for women, as well as patients who were already taking a statin, even though their LDL-C and triglycerides concentrations had already been lowered to within normal limits.

**Table 2:** Medication use in patient groups according to CAD presence.

	Patients with CAD (*n* = 146)	Patients Without CAD (*n* = 20)	*p*-Value
Statin, *n* (%)	78 (53.4)	7 (35)	0.154
Ezetimibe, *n* (%)	7 (4.8)	0 (0)	0.317
ACE inhibitor/ARB, *n* (%)	97 (66.4)	10 (50)	0.212
Beta-blocker, *n* (%)	91 (62.3)	10 (50)	0.333
Calcium channel blocker, *n* (%)	34 (23.3)	4 (20)	0.743
MRA, *n* (%)	9 (6.2)	0 (0)	0.602
Aspirin, *n* (%)	39 (27.3)	1 (5)	0.047
Ranolazine, *n* (%)	7 (4.8)	1 (5)	0.895
Trimetazidine, *n* (%)	11 (7.5)	1 (5)	0.805

Values are numbers with percentages in parentheses. CAD, coronary artery disease; ACE, angiotensin-converting enzyme; ARB, angiotensin II receptor blocker; MRA, mineralocorticoid receptor antagonist.

**Table 3:** Baseline characteristics of patients with CAD grouped according to Gensini score.

	Groups by Gensini Score			*p*-Value
	Group 1 (*n* = 44)	Group 2 (*n* = 36)	Group 3 (*n* = 27)	
Age, years	63 (48–80)	65 (48–82)	70 (49–85)	0.058
Female gender, *n* (%)	20 (45.5)	13 (36.1)	7 (25.9)	0.418
BMI, kg/m^2^	28.68 (22.77–41.03)	29.4 (21.57–38.4)	29.09 (23.64–35.99)	0.633
Smoking, *n* (%)	6 (13.6)	15 (42.9)	13 (48.1)	0.302
Obesity, *n* (%)	17 (40.5)	16 (44.4)	8 (32)	0.713
Arterial hypertension, *n* (%)	40 (90.9)	35 (97.2)	25 (92.6)	0.251
Dyslipidemia, *n* (%)	42 (95.5)	34 (94.4)	25 (92.6)	0.112
Early family history of cardiovascular disease, *n* (%)	9 (24.3)	6 (18.2)	4 (20)	0.312
Systolic BP, mmHG	143 (120–180)	148 (125–170)	138 (116–156)	0.074
Diastolic BP, mmHg	85 (70–100)	89 (68–108)	78 (64–102)	0.272
Heart rate, bpm	66 (54–100)	68 (59–95)	68 (60–80)	0.645
Uric acid, μmol	331 (185–467)	326.5 (263–627)	375 (300–474)	0.241

Values are medians, with minimum and maximum values in parentheses or numbers with percentages in parentheses. BMI, body mass index; BP, blood pressure; bpm, beats per minute; CAD, coronary artery disease. Group 1 consisted of patients with a Gensini score between 0 and 11; Group 2 consisted of patients with a Gensini score between 12 and 35; Group 3 consisted of patients with a Gensini score above 35. Dyslipidemia group consisted of patients with total cholesterol concentration greater than 5 mmol/L or LDL-C concentration greater than 3 mmol/L or triglycerides concentrations greater than 1.7 mmol/L or HDL-C less than 1 mmol/L for men, <1.2 mmol/L for women, as well as patients who were already taking a statin, even though their LDL-C and triglycerides concentrations had already been lowered to within normal limits.

**Table 4:** Medication use in patients with CAD grouped according to Gensini score.

	Groups by Gensini Score			*p*-Value
	Group 1 (*n* = 44)	Group 2 (*n* = 36)	Group 3 (*n* = 27)	
Statin, *n* (%)	25 (56.8)	24 (66.7)	20 (74.1)	0.202
Ezetimibe, *n* (%)	3 (6.8)	2 (5.6)	2 (7.4)	0.816
ACE inhibitor/ARB, *n* (%)	31 (70.5)	30 (83.3)	20 (74.1)	0.318
Beta-blocker, *n* (%)	31 (70.5)	26 (72.2)	19 (70.4)	0.520
Calcium channel blocker, *n* (%)	11 (25)	9 (25)	9 (33.3)	0.864
MRA, *n* (%)	3 (6.8)	3 (8.3)	1 (3.7)	0.898
Aspirin, *n* (%)	12 (27.3)	17 (51.5)	10 (37)	0.461
Ranolazine, *n* (%)	1 (2.3)	2 (5.6)	4 (14.8)	0.558
Trimetazidine, *n* (%)	3 (6.8)	4 (11.1)	4 (14.8)	0.515

Values are numbers with percentages in parentheses. ACE, angiotensin-converting enzyme; ARB, angiotensin II receptor blocker; MRA, mineralocorticoid receptor antagonist; CAD, coronary artery disease. Group 1 consisted of patients with a Gensini score between 0 and 11; Group 2 consisted of patients with a Gensini score between 12 and 35; Group 3 consisted of patients with a Gensini score above 35.

**Table 5:** Baseline characteristics of patients grouped according to CAD-RADS score.

	Groups by CAD-RADS Score				*p*-Value
	Group 1 ^a^ (*n* = 13)	Group 2 ^b^ (*n* = 26)	Group 3 ^c^ (*n* = 21)	Group 4 ^d^ (*n* = 39)	
Age, years	52 (34–79) ^ac,ad^	56 (38–61)	61 (42–80) ^ac^	62 (44–82) ^ad^	<0.001 ^ac,ad^
Female gender, *n* (%)	7 (53.8)	11 (42.3)	13 (61.9)	13 (33.3)	0.168
BMI, kg/m^2^	29 (22.14–38.05)	27.47 (21.65–35.19)	29.67 (19.57–40.12)	29.09 (19.96–35.36)	0.979
Smoking, *n* (%)	3 (23.1)	6 (23.1)	5 (23.8)	14 (35.9)	0.606
Obesity, *n* (%)	5 (38.5)	11 (42.3)	8 (38.1)	12 (32.4)	0.964
Arterial hypertension, *n* (%)	10 (83.3) ^ab^	14 (53.8) ^ab,bc,bd^	19 (90.5) ^bd,bc^	35 (89.7) ^bd^	0.002 ^ab^ 0.004 ^bc^ 0.023 ^bd^
Early family history of cardiovascular disease, *n* (%)	1 (7.7)	4 (15.4)	5 (25)	7 (22.6)	0.565
Systolic BP, mmHG	142 (116–180)	128 (116–185)	140 (120–176)	147 (120–170)	0.124
Diastolic BP, mmHg	88 (78–106)	87 (79–120)	87 (70–101)	88 (64–105)	0.705
Heart rate, bpm	70 (56–79)	68 (50–100)	63 (58–86)	68 (56–86)	0.473
Uric acid, μmol	351 (180–459)	310 (154–389)	358 (189–485)	374 (231–474)	0.477

Values are medians, with minimum and maximum values in parentheses or numbers with percentages in parentheses. BMI, body mass index; BP, blood pressure; bpm, beats per minute. Group 1 is patients with CAD-RADS 0—no plaque or stenosis; Group 2 is patients with CAD-RADS 1—minimal stenosis: 1 to 24% or CAD-RADS 2—mild stenosis: 25 to 49%; Group 3 is patients with CAD-RADS 3—moderate stenosis: 50 to 69%; Group 4 is patients with CAD-RADS 4—severe stenosis: 70 to 99%, or a left main ≥ 50%, or three-vessel obstructive disease ≥ 70% or CAD-RADS 5—at least one completely occluded coronary artery. The superscript letter “a” indicates Group 1st, “b”—Group 2nd, “c”—Group 3rd, “d”—Group 4th. Marker “ab” indicates a significance value between 1st and 2nd groups; “ac”—between 1st and 3rd groups; “ad”—between 1st and 4th groups; “bc”—between 2nd and 3rd groups; “bd”—between 2nd and 4th groups. Dyslipidemia group consisted of patients with total cholesterol concentration greater than 5 mmol/L or LDL-C concentration greater than 3 mmol/L or triglycerides concentrations greater than 1.7 mmol/l or HDL-C less than 1 mmol/L for men, <1.2 mmol/L for women, as well as patients who were already taking a statin, even though their LDL-C and triglycerides concentrations had already been lowered to within normal limits.

**Table 6:** Medication use in patients grouped by CAD-RADS score.

	Groups by CAD-RADS Score				*p*-Value
	Group 1 ^a^ (*n* = 13)	Group 2 ^b^ (*n* = 26)	Group 3 ^c^ (*n* = 21)	Group 4 ^d^ (*n* = 39)	
Statin, *n* (%)	3 (23.1) ^ad^	8 (30.8) ^bd^	8 (38.1) ^cd^	24 (61.5) ^ad,bd,cd^	0.003 ^ad^ 0.023 ^bd^ 0.025 ^cd^
ACE inhibitor/ARB, *n* (%)	7 (53.8)	8 (30.8) ^bc,bd^	14 (66.7) ^bc^	27 (69.2) ^bd^	0.015 ^bc^ 0.021 ^bd^
Beta-blocker, *n* (%)	5 (38.5) ^ac^	7 (26.9) ^bc^	17 (81) ^ac, bc^	24 (61.5)	<0.001 ^ac,bc^
Aspirin, *n* (%)	0 (0) ^ad^	0 (0) ^bd^	2 (9.5) ^cd^	15 (39.5) ^ad,bd,cd^	<0.001 ^ad,bd,cd^

Values are medians, with minimum and maximum values in parentheses or numbers with percentages in parentheses. ACE, angiotensin-converting enzyme; ARB, angiotensin II receptor blocker. Group 1 consisted of patients with no coronary stenosis (CAD-RADS 0); Group 2 consisted of patients with minimal and mild stenosis (CAD-RADS 1 and 2); Group 3 consisted of patients with moderate stenosis (CAD-RADS 3); Group 4 consisted of patients with significant stenosis (CAD-RADS 4 and 5). The superscript letter “a” indicates Group 1st, “b”—Group 2nd, “c”—Group 3rd, “d”—Group 4th. Marker “ac”—between 1st and 3rd groups; “ad”—between 1st and 4th groups; “bc”—between 2nd and 3rd groups; “bd”—between 2nd and 4th groups; “cd”—between 3rd and 4th groups.

**Table 7:** Comparison of inflammation and total blood count readings in patients with and without CAD.

	Patients with CAD (*n* = 146)	Patients Without CAD (*n* = 20)	*p*-Value
Hs-CRP, mg/L	1.28 (0.12–6.47)	0.68 (0.12–2.22)	0.048
Platelet count, ×10^9^/L	220 (114–367)	275 (178–337)	0.019
Erythrocyte count, ×10^12^/L	4.67 (3.40–5.55)	4.81 (4.11–5.3)	0.037
Leukocyte count, ×10^9^/L	6.4 (3.70–10.27)	6.27 (4.10–7.60)	0.148
Neutrophil count, ×10^9^/L	3.54 (1.80–7.70)	3.23 (1.90–4.80)	0.021
Neutrophil count, %	57.50 (32.60–75.90)	49.15 (43.30–64.60)	0.002
Lymphocyte count, ×10^9^/L	2.00 (1.10–4.12)	2.28 (1.60–3.30)	0.032
Lymphocyte count, %	31.20 (14.60–48.30)	37.55 (25.70–46.30)	<0.001
Monocyte count, ×10^9^/L	0.50 (0.24–0.88)	0.50 (0.30–0.68)	0.052
Monocyte count, %	8.37 (4.20–14.50)	7.55 (6.20–14.80)	0.115
MPV, fl	9.00 (6.40–12.80)	9.15 (6.90–11.90)	0.407

Values are medians, with minimum and maximum values in parentheses. CAD, coronary artery disease; hs-CRP, high-sensitivity C-reactive protein; MPV, mean platelet volume.

**Table 8:** Calculated total blood cell counts readings in patients with and without CAD.

	Patients with CAD (*n* = 146)	Patients Without CAD (*n* = 20)	*p*-Value
PLR, ×10^9^/L	112.61 (48.06–224.29)	116.09 (81.78–186.25)	0.585
NLR, ×10^9^/L	1.91 (0.67–5.13)	1.30 (0.96–2.56)	<0.001
NMR, ×10^9^/L	7.0 (3.0–15.40)	6.10 (3.17–10.25)	0.666
LMR, ×10^9^/L	4.00 (1.38–9.50)	4.37 (2.67–6.61)	<0.001
MLR, ×10^9^/L	0.25 (0.11–0.73)	0.23 (0.15–0.38)	<0.001
SIRI, ×10^9^/L	0.93 (0.33–2.57)	0.70 (0.34–1.14)	<0.001
MHR	0.38 (0.11–1.0)	0.33 (0.13–0.85)	0.062

Values are medians, with minimum and maximum values in parentheses. CAD, coronary artery disease; PLR, platelet-to-lymphocyte ratio; NLR, neutrophil-to-lymphocyte ratio; NMR, neutrophil-to-monocyte ratio; LMR, lymphocyte-to-monocyte ratio; MLR, monocyte-to-lymphocyte ratio; SIRI, systemic inflammation response index; MHR, monocyte-to-high-density- lipoprotein ratio.

**Table 9:** Inflammation and total blood count readings in patients grouped by Gensini score.

	Groups by Gensini Score			*p*-Value
	Group 1 ^a^ (*n* = 44)	Group 2 ^b^ (*n* = 36)	Group 3 ^c^ (*n* = 27)	
Hs-CRP, mg/L	1.81 (0.12–6.47)	1.32 (0.28–3.82)	3.21 (0.4–4.42)	0.117
Platelet count, ×10^9^/L	217 (114–291)	219 (152–318)	225 (193–314)	0.442
Erythrocyte count, ×10^12^/L	4.59 (4.04–5.55)	4.62 (4.1–5.01)	4.95 (3.40–5.23)	0.092
Leukocyte count, ×10^9^/L	6.07 (4.23–9.1)	6.4 (4.93–10.2)	7.1 (5.4–8.4)	0.298
Neutrophil count, ×10^9^/L	2.89 (2.08–6.0) ^ab,ac^	3.54 (1.8–7.7)	4.52 (3.1–4.9) ^ac^	0.006 ^ab^ 0.003 ^ac^
Neutrophil count, %	53.5 (41.1–66.8) ^ab,ac^	57.5 (32.6–75.9)	60.8 (58.1–63.7) ^ac^	<0.001 ^ab,ac^
Lymphocyte count, ×10^9^/L	2 (1.2–4.12)	2.28 (1.1–3.42)	1.96 (1.4–2.7)	0.056
Lymphocyte count, %	36.8 (21.9–46.3) ^ab,ac^	31.2 (14.6–48.3) ^ab^	27.6 (21.4–32.1) ^ac^	<0.001 ^ab,ac^
Monocyte count, ×10^9^/L	1.81 (0.12–6.47)	0.5 (0.24–0.65)	0.6 (0.5–0.8)	0.522
Monocyte count, %	217 (114–291)	6.6 (4.2–10.1)	9.3 (6.4–13.4)	0.644
MPV, fl	8.6 (6.6–11.2)	8.8 (6.4–12.8)	8.5 (4.5–11.4)	0.900

Values are medians, with minimum and maximum values in parentheses. hs-CRP, high-sensitivity C-reactive protein; MPV, mean platelet volume. Group 1 consisted of patients with a Gensini score between 0 and 11; Group 2 consisted of patients with a Gensini score between 12 and 35; Group 3 consisted of patients with a Gensini score above 35. The superscript letter “a” indicates Group 1st, “b”—Group 2nd, “c”—Group 3rd. Marker “ab” indicates a significance value between 1st and 2nd groups; “ac”—between 1st and 3rd groups.

**Table 10:** Ratio of total blood cell counts in patients grouped by Gensini score.

	Groups by Gensini Score			*p*-Value
	Group 1 ^a^ (*n* = 44)	Group 2 ^b^ (*n* = 36)	Group 3 ^c^ (*n* = 27)	
PLR, ×10^9^/L	99.55 (48.06–194) ^ac^	96.07 (63.74–190)	114.80 (87.73–224.29) ^ac^	0.005 ^ac^
NLR, ×10^9^/L	1.48 (0.89–3.0) ^ab,ac^	1.98 (0.67–5.13) ^ab^	2.21 (1.81–2.93) ^ac^	<0.001 ^ab,ac^
NMR, ×10^9^/L	5.78 (4.05–8.6)	8.66 (3.0–15.4)	6.86 (5.16–9.8)	0.523
LMR, ×10^9^/L	4.33 (2.0–5.8) ^ac^	4.6 (2.75–9.5)	3.14 (1.88–5.4) ^ac^	<0.001 ^ac^
MLR, ×10^9^/L	0.23 (0.17–0.50) ^ac^	0.2 (0.11–0.36) ^bc^	0.32 (0.19–0.53) ^ac,bc^	<0.001 ^ac,bc^
SIRI, ×10^9^/L	0.72 (0.44–2.4) ^ab,ac^	0.89 (0.33–2.57) ^ab^	1.33 (0.91–2.35) ^ac,bc^	<0.001 ^ab,ac,bc^
MHR	0.42 (0.22–1)	0.33 (0.13–0.66)	0.57 (0.42–0.67)	0.585

Values are medians, with minimum and maximum values in parentheses. PLR, platelet-to-lymphocyte ratio; NLR, neutrophil-to-lymphocyte ratio; NMR, neutrophil-to-monocyte ratio; LMR, lymphocyte-to-monocyte ratio; MLR, monocyte-to-lymphocyte ratio; SIRI, systemic inflammation response index; MHR, monocyte-to-high-density-lipoprotein ratio. Group 1 consisted of patients with a Gensini score between 0 and 11; Group 2 consisted of patients with a Gensini score between 12 and 35; Group 3 consisted of patients with a Gensini score above 35. The superscript letter “a” indicates Group 1st, “b”—Group 2nd, “c”—Group 3rd. Marker “ab” indicates a significance value between 1st and 2nd groups; “ac”—between 1st and 3rd groups; “bc”—between 2nd and 3rd groups.

**Table 11:** Inflammation and total blood counts readings in patients grouped according to CAD-RADS classification.

	Groups by CAD-RADS Score				*p*-Value
	Group 1 ^a^ (*n* = 13)	Group 2 ^b^ (*n* = 26)	Group 3 ^c^ (*n* = 21)	Group 4 ^d^ (*n* = 39)	
Hs-CRP, mg/L	0.75 (0.43–2.22)	0.84 (0.15–3.22)	1.51 (0.16–3.79)	1.47 (0.12–6.47)	0.336
Platelet count, ×10^9^/L	282 (202–337) ^ac^	215.5 (155–290)	200.9 (114–318) ^ac^	228 (151–367)	0.030 ^ac^
Erythrocyte count, ×10^12^/L	4.78 (4.11–5.27)	4.74 (3.59–5.49)	4.71 (4.27–5.33)	4.64 (3.4–5.55)	0.887
Leukocyte count, ×10^9^/L	6.6 (5.2–7.6)	5.94 (3.7–7.5) ^bd^	6.85 (4.3–10.27)	7.54 (4.93–10.2) ^bd^	0.002 ^bd^
Neutrophil count, ×10^9^/L	3.25 (2.3–4.8)	3.18 (2.1–3.86) ^bd^	4.07 (2.5–6.77)	4.0 (2.79–7.7) ^bd^	0.001 ^bd^
Neutrophil count, %	49.1 (43.3–64.6) ^ad^	55.35 (43.6–69.2)	58.88 (47.5–73.4)	59.0 (41.1–75.9) ^ad^	0.023 ^ad^
Lymphocyte count, ×10^9^/L	2.4 (1.6–3.3)	1.8 (1.1–3.35)	1.99 (1.1–3.64)	2.2 (1.4–4.12)	0.208
Lymphocyte count, %	37.6 (25.7–46.3) ^ad^	32.95 (20.4–46.6)	28.59 (20.3–42.1)	28.6 (14.6–46.3) ^ad^	0.003 ^ad^
Monocyte count, ×10^9^/L	0.5 (0.34–0.68) ^ad,bd^	0.51 (0.3–0.81) ^bd^	0.54 (0.37–0.88)	0.6 (0.24–0.81) ^ad,bd^	<0.001 ^ad,bd^
Monocyte count, %	6.9 (6.2–10.2) ^ad^	8.41 (6.8–12.7)	8.65 (4.8–14.5)	9.1 (4.2–11.0) ^ad^	0.029 ^ad^
MPV, fl	8.7 (6.9–10.9)	9.15 (7.4–12.6)	9.05 (7.7–10.6)	8.9 (6.4–11.2)	0.623

Values are medians, with minimum and maximum values in parentheses. hs-CRP, high-sensitivity C-reactive protein; MPV, mean platelet volume. Group 1 consisted of patients with no coronary stenosis (CAD-RADS 0); Group 2 consisted of patients with minimal and mild stenosis (CAD-RADS 1 and 2); Group 3 consisted of patients with moderate stenosis (CAD-RADS 3); Group 4 consisted of patients with significant stenosis (CAD-RADS 4 and 5). The superscript letter “a” indicates Group 1st, “b”—Group 2nd, “c”—Group 3rd, “d”—Group 4th. Marker “ac”—between 1st and 3rd groups; “ad”—between 1st and 4th groups; “bd”—between 2nd and 4th groups.

**Table 12:** Comparison of the ratio of total blood cell counts in patients grouped according to CAD-RADS classification.

	Groups by CAD-RADS Score				*p*-Value
	Group 1 ^a^ (*n* = 13)	Group 2 ^b^ (*n* = 26)	Group 3 ^c^ (*n* = 21)	Group 4 ^d^ (*n* = 39)	
PLR, ×10^9^/L	120.36 (81.78–186.25)	119.54 (50.15–193.34)	109.64 (73.08–180.0)	119.47 (48.06–224.29)	0.360
NLR, ×10^9^/L	1.29 (0.96–2.56) ^ad^	1.66 (0.93–3.28)	2.07 (1.13–3.59)	2.09 (0.89–5.14) ^ad^	0.007 ^ad^
NMR, ×10^9^/L	6.45 (4.73–10.25)	6.57 (3.67–9.0)	7.05 (4.17–15.27)	6.86 (4.51–15.4)	0.937
LMR, ×10^9^/L	5.08 (3.68–6.61) ^ad^	3.95 (1.84–5.58) ^bd^	4.17 (1.38–5.71)	3.17 (2.34–9.5) ^ad,bd^	<0.001 ^ad^ 0.046 ^bd^
MLR, ×10^9^/L	0.20 (0.15–0.27) ^ad^	0.25 (0.18–0.55)	0.24 (0.18–0.73)	0.32 (0.11–0.43) ^ad^	<0.001 ^ad^
SII, ×10^9^/L	312.29 (265.46–763.62)	342.73 (156.97–674.55)	432.72 (222.75–876.63)	453.0 (175.41–1314.13)	0.113
MHR	0.28 (0.13–0.85) ^ad^	0.36 (0.11–0.72) ^bd^	0.4 (0.22–0.65)	0.48 (0.13–1.0) ^ad,bd^	0.010 ^ad^ 0.036 ^bd^

Values are medians, with minimum and maximum values in parentheses. PLR, platelet-to-lymphocyte ratio; NLR, neutrophil-to-lymphocyte ratio; NMR, neutrophil-to-monocyte ratio; LMR, lymphocyte-to-monocyte ratio; MLR, monocyte-to-lymphocyte ratio; SII, systemic immune-inflammation index; MHR, monocyte-to-high-density-lipoprotein ratio. Group 1 consisted of patients with no coronary stenosis (CAD-RADS 0); Group 2 consisted of patients with minimal and mild stenosis (CAD-RADS 1 and 2); Group 3 consisted of patients with moderate stenosis (CAD-RADS 3); Group 4 consisted of patients with significant stenosis (CAD-RADS 4 and 5). The superscript letter “a” indicates Group 1st, “b”—Group 2nd, “c”—Group 3rd, “d”—Group 4th. Marker “ad”—between 1st and 4th groups; “bd”—between 2nd and 4th groups.

**Table 13:** Correlations between echocardiography and total blood count readings.

Readings	PLT	Neutrophil Percentage	Neutrophil Count	Lymphocyte Percentage	Lymphocyte Count
LVEF	*r* = 0.130, *p* = 0.100	***r* = −0.236, *p* = 0.002**	***r* = −0.232, *p* = 0.003**	***r* = 0.236** ***p* = 0.003**	*r* = 0.121, *p* = 0.126
LVEDD	***r* = −0.248,** ***p* = 0.002**	***r* = 0.187, *p* = 0.021**	***r* = 0.173** **, *p* = 0.032**	***r* = −0.239,** ***p* = 0.003**	***r* = −0.160, *p* = 0.048**
IVS thickness	*r* = −0.129, *p* = 0.110	***r* = 0.163, *p* = 0.043**	*r* = 0.139, *p* = 0.087	*r* = −0.112, *p* = 0.168	*r* = −0.036, *p* = 0.654
LVM	***r* = −0.272,** ***p* < 0.001**	***r* = 0.231, *p* = 0.004**	***r* = 0.179, *p* = 0.027**	***r* = −0.218,** ***p* = 0.007**	*r* = −0.123, *p* = 0.131
LVMi	***r* = −0.261,** ***p* = 0.001**	***r* = 0.218, *p* = 0.007**	*r* = 0.128, *p* = 0.115	***r* = −0.181,** ***p* = 0.025**	*r* = −0.127, *p* = 0.119
LA diameter	***r* = −0.270,** ***p* < 0.001**	*r* = 0.138, *p* = 0.094	*r* = 0.126, *p* = 0.125	***r* = −0.171,** ***p* = 0.037**	*r* = −0.098, *p* = 0.073
LA volume	***r* = −0.350,** ***p* < 0.001**	*r* = 0.192, *p* = 0.080	*r* = 0.108, *p* = 0.329	*r* = −0.195, *p* = 0.075	*r* = −0.197, *p* = 0.073
LA volume index	***r* = −0.282,** ***p* = 0.015**	***r* = 0.230, *p* = 0.049**	*r* = 0.140, *p* = 0.234	***r* = −0.242,** ***p* = 0.038**	*r* = −0.211, *p* = 0.071
e′_lat_	***r* = 0.221,** ***p* = 0.029**	***r* = −0.216, *p* = 0.033**	*r* = −0.126, *p* = 0.215	***r* = 0.232,** ***p* = 0.021**	*r* = 0.197, *p* = 0.052
e′_sep_	***r* = 0.362,** ***p* < 0.001**	***r* = −0.250, *p* = 0.014**	*r* = −0.139, *p* = 0.177	***r* = 0.316,** ***p* = 0.002**	***r* = 0.273, *p* = 0.007**
e′	***r* = 0.336,** ***p* < 0.001**	***r* = −0.249, *p* = 0.013**	*r* = −0.153, *p* = 0.132	***r* = 0.293,** ***p* = 0.003**	***r* = 0.242, *p* = 0.016**
E/e′	***r* = −0.228,** ***p* = 0.024**	*r* = 0.072, *p* = 0.484	*r* = 0.108, *p* = 0.292	*r* = −0.131, *p* = 0.198	*r* = −0.110, *p* = 0.281

PLT, platelets; LVEF, left ventricular ejection fraction; LVEDD, left ventricular end-diastolic diameter; IVS, intraventricular septal; LVM, left ventricular mass; LVMi, left ventricular mass index; LA, left atrium; e′_lat_, late diastolic velocity measured in the lateral mitral annulus; e′_sep_, late diastolic velocity measured in the septal mitral annulus; e′, average e′_sep_ and e′_lat_; E/e′, ratio of peak early diastolic velocity (E) and e′. Significant Spearman’s correlations and *p*-values are in bold.

**Table 14:** Correlations between echocardiography and total blood count calculated readings.

Readings	LVEF	LVEDD	LA Diameter	e′	e′_sep_
NLR	***r* = −0.230,** ***p* = 0.003**	***r* = 0.229,** ***p* = 0.004**	*r* = 0.149, *p* = 0.071	***r* = −0.291,** ***p* = 0.004**	***r* = −0.305,** ***p* = 0.003**
MLR	***r* = −0.186,** ***p* = 0.018**	*r* = 0.016, *p* = 0.842	***r* = 0.269,** ***p* < 0.001**	***r* = −0.202,** ***p* = 0.046**	***r* = −0.302,** ***p* = 0.003**
LMR	***r* = 0.186,** ***p* = 0.018**	***r* = −0.263,** ***p* = 0.001**	***r* = −0.270,** ***p* < 0.001**	***r* = 0.203,** ***p* = 0.045**	***r* = 0.302,** ***p* = 0.003**
SIRI	***r* = −0.241,** ***p* = 0.002**	***r* = 0.260,** ***p* = 0.001**	***r* = 0.252,** ***p* = 0.002**	***r* = −0.235,** ***p* = 0.020**	***r* = −0.304,** ***p* = 0.003**
MHR	*r* = −0.113, *p* = 0.179	***r* = 0.180,** ***p* = 0.039**	***r* = 0.217,** ***p* = 0.014**	*r* = −0.064, *p* = 0.550	*r* = −0.125, *p* = 0.248

LVEF, left ventricular ejection fraction; LVEDD, left ventricular end-diastolic diameter; LA, left atrium; e′_sep_, late diastolic velocity were measured in the septal mitral annulus; e′, average e′_sep_ and e′_lat_; NLR, neutrophil-to-lymphocyte ratio; MLR, monocyte-to-lymphocyte ratio, LMR, lymphocyte-to-monocyte ratio; NMR, neutrophil-to-monocyte ratio; PLR, platelet-to-lymphocyte ratio; SIRI, systemic inflammation response index; MHR, monocyte-to-high-density-lipoprotein ratio. Significant Spearman’s correlations and *p*-values are in bold.

**Table 15:** Correlations between lipid and total blood count readings.

Readings	Total Cholesterol Concentration	LDL	HDL	Lp(a)
Leukocyte count	*r* = −0.079, *p* = 0.347	*r* = −0.100, *p* = 0.226	***r* = −0.206,** ***p* = 0.013**	***r* = −0.290,** ***p* = 0.012**
Neutrophil count	*r* = −0.078, *p* = 0.352	*r* = −0.070, *p* = 0.401	***r* = −0.165,** ***p* = 0.049**	*r* = −0.153, *p* = 0.194
Monocyte count	***r* = −0.207,** ***p* = 0.013**	*r* = −0.162, *p* = 0.050	***r* = −0.348,** ***p* < 0.001**	***r* = −0.288,** ***p* = 0.013**
Monocyte, %	*r* = −0.116, *p* = 0.167	*r* = −0.044, *p* = 0.601	***r* = −0.243,** ***p* = 0.003**	*r* = −0.194, *p* = 0.097

Lp(a), lipoprotein (a). Significant Spearman’s correlations and *p*-values are in bold.

**Table 16:** Correlations between lipid and calculated total blood count readings.

Readings	Total Cholesterol Concentration	LDL	HDL	TG	ApoB	Lp(a)
PLR	*r* = 0.014, *p* = 0.867	*r* = 0.024, *p* = 0.771	***r* = 0.176,** ***p* = 0.035**	*r* = −0.051, *p* = 0.554	*r* = 0.040, *p* = 0.741	*r* = 0.206, *p* = 0.079
LMR	***r* = 0.220,** ***p* = 0.008**	*r* = 0.156, *p* = 0.059	***r* = 0.264,** ***p* = 0.001**	*r* = 0.025, *p* = 0.775	*r* = 0.222, *p* = 0.061	*r* = 0.170, *p* = 0.147
MLR	***r* = −0.221,** ***p* = 0.008**	*r* = −0.157, *p* = 0.058	***r* = −0.265,** ***p* = 0.001**	*r* = −0.025, *p* = 0.774	*r* = −0.222, *p* = 0.060	*r* = −0.171, *p* = 0.146
SIRI	***r* = −0.206,** ***p* = 0.013**	*r* = −0.159, *p* = 0.054	***r* = −0.262,** ***p* = 0.002**	*r* = 0.051, *p* = 0.548	***r* = −0.261,** ***p* = 0.027**	*r* = −0.158, *p* = 0.179
MHR	***r* = −0.353,** ***p* < 0.001**	***r* = −0.245,** ***p* = 0.003**	***r* = −0.801,** ***p* < 0.001**	***r* = 0.170,** ***p* = 0.046**	*r* = −0.209, *p* = 0.083	***r* = −0.364,** ***p* = 0.002**

TG, triglycerides; apoB, apolipoprotein B; Lp(a), lipoprotein (a); PLR, platelet-to-lymphocyte ratio; LMR, lymphocyte-to-monocyte ratio; MLR, monocyte-to-lymphocyte ratio; SIRI, systemic inflammation response index; MHR, monocyte-to-high-density-lipoprotein ratio. Significant Spearman’s correlations and *p*-values are in bold.

**Table 17:** Correlations between baseline and total blood count and inflammation readings.

Readings	Age	Smoking	BMI	Obesity	Early History of CVD	DBP	AH
Leukocyte count	*r* = −0.190, *p* = 0.083	*r* = 0.156, *p* = 0.158	***r* = 0.239,** ***p* = 0.031**	*r* = 0.146, *p* = 0.193	*r* = 0.197, *p* = 0.075	*r* = 0.199, *p* = 0.070	***r* = 0.247,** ***p* = 0.024**
Neutrophil count	*r* = −0.095, *p* = 0.392	*r* = 0.099, *p* = 0.372	*r* = 0.151, *p* = 0.174	*r* = 0.023, *p* = 0.839	***r* = 0.252,** ***p* = 0.022**	*r* = 0.105, *p* = 0.341	***r* = 0.222,** ***p* = 0.042**
Monocyte count	*r* = 0.066, *p* = 0.548	*r* = 0.182, *p* = 0.099	*r* = 0.075, *p* = 0.504	*r* = −0.031, *p* = 0.783	*r* = −0.006, *p* = 0.955	*r* = 0.092, *p* = 0.406	***r* = 0.219,** ***p* = 0.045**
PLT	***r* = −0.222,** ***p* = 0.043**	***r* = −0.224,** ***p* = 0.042**	*r* = −0.042, *p* = 0.710	*r* = −0.007, *p* = 0.951	*r* = 0.087, *p* = 0.433	*r* = −0.021, *p* = 0.847	*r* = −0.065, *p* = 0.558
PLR	*r* = 0.074, *p* = 0.518	***r* = −0.311,** ***p* = 0.006**	***r* = −0.258,** ***p* = 0.023**	*r* = −0.187, *p* = 0.106	*r* = 0.046, *p* = 0.691	***r* = −0.254,** ***p* = 0.024**	*r* = −0.073, *p* = 0.521
SII	*r* = 0.037, *p* = 0.742	*r* = −0.197, *p* = 0.076	*r* = −0.109, *p* = 0.335	*r* = −0.123, *p* = 0.278	***r* = 0.253,** ***p* = 0.021**	*r* = −0.130, *p* = 0.241	*r* = 0.173, *p* = 0.118
Hs-CRP	*r* = −0.146, *p* = 0.332	*r* = 0.210, *p* = 0.160	***r* = 0.373,** ***p* = 0.011**	***r* = 0.331,** ***p* = 0.025**	*r* = −0.017, *p* = 0.912	*r* = 0.235, *p* = 0.116	*r* = 0.073, *p* = 0.630

BMI, body mass index; CVD, cardiovascular disease; DBP, diastolic blood pressure; AH, arterial hypertension; PLT, platelets; PLR, platelet-to-lymphocyte ratio; SII, systemic immune-inflammation index; Hs-CRP, high sensitivity C-reaction protein. Significant Spearman’s correlations and *p*-values are in bold.

**Table 18:** Drug usage correlations with lipid and uric acid readings.

Readings	Beta-Blocker	MRA	ACEi/ARB	Statin	Trimetazidine/Ranolazine
Total cholesterol	*r* = −0.074, *p* = 0.379	***r* = −0.174, *p* = 0.036**	***r* = −0.191, *p* = 0.022**	***r* = −0.212, *p* = 0.010**	***r* = −0.175, *p* = 0.035**
LDL-C	*r* = −0.063, *p* = 0.445	***r* = −0.206, *p* = 0.012**	***r* = −0.210, *p* = 0.010**	***r* = −0.183, *p* = 0.026**	*r* = −0.130, *p* = 0.115
HDL-C	***r* = −0.227, *p* = 0.006**	*r* = −0.150, *p* = 0.071	*r* = −0.152, *p* = 0.068	***r* = −0.288, *p* < 0.001**	*r* = −0.145, *p* = 0.083
TG	*r* = 0.128, *p* = 0.132	***r* = 0.196, *p* = 0.020**	*r* = −0.009, *p* = 0.920	*r* = 0.057, *p* = 0.507	*r* = −0.050, *p* = 0.555
ApoB	***r* = −0.341, *p* = 0.003**	***r* = −0.273, *p* = 0.019**	*r* = −0.214, *p* = 0.069	*r* = −0.149, *p* = 0.207	***r* = −0.360, *p* = 0.002**
Lp(a)	*r* = −0.161, *p* = 0.166	*r* = −0.007, *p* = 0.954	*r* = −0.078, *p* = 0.505	***r* = −0.261, *p* = 0.024**	***r* = −0.296, *p* = 0.010**

LDL-C, low-density lipoprotein cholesterol; HDL-C, high-density lipoprotein cholesterol; TG, triglycerides; apoB, apolipoprotein B; Lp(a), lipoprotein (a); MRA, mineralocorticoid receptor antagonist; ACEi, angiotensin-converting enzyme inhibitor; ARB, angiotensin II receptor blocker. Significant Spearman’s correlations and *p*-values are in bold.

**Table 19:** Correlations between the total blood readings and drug usage.

Readings	Beta-Blocker	ACEi/ARB	Statin	Aspirin
Lymphocyte, %	***r* = −0.186, *p* = 0.016**	***r* = −0.213, *p* = 0.006**	***r* = −0.267, *p* < 0.001**	***r* = −0.282, *p* < 0.001**
Neutrophil count	*r* = 0.139, *p* = 0.075	***r* = 0.176, *p* = 0.024**	***r* = 0.194,** ***p* = 0.012**	***r* = 0.247, *p* = 0.002**
Neutrophil, %	*r* = 0.145, *p* = 0.064	***r* = 0.173, *p* = 0.026**	***r* = 0.202,** ***p* = 0.009**	***r* = 0.205, *p* = 0.009**
NLR	***r* = 0.176, *p* = 0.024**	***r* = 0.203, *p* = 0.009**	***r* = 0.253,** ***p* = 0.001**	***r* = 0.261, *p* < 0.001**
LMR	***r* = −0.157, *p* = 0.044**	***r* = −0.162, *p* = 0.038**	***r* = −0.244, *p* = 0.002**	***r* = −0.183, *p* = 0.020**
MLR	***r* = 0.157, *p* = 0.044**	***r* = 0.161, *p* = 0.039**	***r* = 0.243,** ***p* = 0.002**	***r* = 0.183, *p* = 0.020**
SII	*r* = 0.088, *p* = 0.262	*r* = 0.152, *p* = 0.051	*r* = 0.126, *p* = 0.108	***r* = 0.254, *p* = 0.001**
SIRI	***r* = 0.188, *p* = 0.016**	***r* = 0.218, *p* = 0.005**	***r* = 0.248,** ***p* = 0.001**	***r* = 0.258, *p* < 0.001**
MHR	***r* = 0.183, *p* = 0.028**	*r* = 0.121, *p* = 0.148	***r* = 0.246,** ***p* = 0.003**	***r* = 0.175, *p* = 0.038**

ACEi, angiotensin-converting enzyme inhibitor; ARB, angiotensin II receptor blocker; NLR, neutrophil-to-lymphocyte ratio; LMR, lymphocyte-to-monocyte ratio; MLR, monocyte-to-lymphocyte ratio; SII, systemic immune-inflammation index; SIRI, systemic inflammation response index; MHR, monocyte-to-high-density-lipoprotein ratio. Significant Spearman’s correlations and *p*-values are in bold.

**Table 20:** Literature findings of relationship between calculated total blood count readings, severity of coronary atherosclerosis, and presence of CAD.

No.	Reference	Investigated Patients	Sample Size (Number; *n*)	Correlations Between Investigated Readings and CAD Severity and Differences Between CAD and Non-CAD Groups			
				SII	NLR	PLR	MLR
1.	Candemir M. et al., 2021 [3]	Patients with SAP (patients with diabetes were included)	669	Spearman’s Rho = 0.630; *p* < 0.001	Spearman’s Rho = 0.557; *p* < 0.001	Spearman’s Rho = 0.479; *p* < 0.001	Spearman’s Rho = 0.234; *p* < 0.001
2.	Ye Z., et al., 2022 [9]	Patients with CAD, stroke, PAL, VT, CSVD	152,996	HR: 1.39, 95% CI: 1.20–1.61, *p* < 0.001	-	-	-
3.	Zhao Z., et al., 2023 [15]	Patients with CAD (patients with diabetes were included)	284	Value in patients with CAD compared to non-CAD group 696.00 vs. 544.82, *p* = 0.002	Value in patients with CAD compared to non-CAD group 3.25 vs. 2.45, *p* < 0.001		Value in patients with CAD compared to non-CAD group 0.31 vs. 0.26, *p* = 0.004
4.	Urbanowicz T., et al., 2023 [16]	Patients with CAD, except patients with ACS and co-existing hematological, rheumatic diseases, and oncological history	256	OR: 1, 95% CI: 0.99–1, *p* = 0.416	OR: 1.23, 95% CI: 0.86–1.76, *p* = 0.253	-	-
5.	Dai X.T., et al., 2022 [19]	Patients with CAD (control and CSFP groups) (patients with diabetes were included)	256	CSFP group 409.7 ± 17.7 vs. Control group 396.7 ± 12.7, *p* < 0.001	CSFP group 1.74 ± 0.09 vs. Control group 1.71 ± 0.12, *p* = 0.034	-	-
6.	Liu Y., et al., 2021 [42]	Patients with suspected CAD (patients with diabetes were included)	395	Spearman’s Rho = 0.538, *p <* 0.001	Spearman’s Rho = 0.490, *p* < *0.001*	Spearman’s Rho = 0.352, *p <* 0.001	-
7.	Ji, H., et al., 2017 [31]	Patients with suspected CAD (patients with diabetes were included)	543	-	No CAD group—1.87 (1.42–2.36) vs. CAD group—2.47 (1.86–3.34), *p* < 0.001	-	No CAD group—0.16 (0.13–0.21) vs. CAD group—0.23 (0.17–0.30), significant association with the Syntax score (*r* = 0.437) *p* < 0.001

SII, systemic immune-inflammation index; NLR, neutrophil-to-lymphocyte ratio; PLR, platelet-to-lymphocyte ratio; MLR, monocyte-to-lymphocyte ratio; SAP, stable angina pectoris; “-“, means that this indicator was not assessed in the study; no, number; CAD, coronary artery disease; PAL, peripheral arterial disease; VT, venous thrombosis; CSVD, cerebral small vessel disease; HR, hazard ratio; ACS, acute coronary syndrome; OR, odds ratio; CSFP, coronary slow flow phenomenon; control group, with normal coronary blood flow.

## Data Availability

The raw data supporting the conclusions of this article will be made available by the authors on request.
